# Senescence‐associated tissue microenvironment promotes colon cancer formation through the secretory factor GDF15

**DOI:** 10.1111/acel.13013

**Published:** 2019-08-06

**Authors:** Yuna Guo, Jessica L. Ayers, Kelly T. Carter, Ting Wang, Sean K. Maden, Darwin Edmond, Polly Newcomb P, Christopher Li, Cornelia Ulrich, Ming Yu, William M. Grady

**Affiliations:** ^1^ Clinical Research Division Fred Hutchinson Cancer Research Center Seattle Washington; ^2^ University of Washington Seattle Washington; ^3^ Public Health Sciences Division Fred Hutchinson Cancer Research Center Seattle Washington; ^4^ Department of Population Health Sciences Huntsman Cancer Institute Salt Lake City Utah; ^5^ Department of Internal Medicine University of Washington School of Medicine Seattle Washington

**Keywords:** colon organoids, colorectal cancer, GDF15, microenvironment, senescence

## Abstract

The risk of colorectal cancer (CRC) varies between people, and the cellular mechanisms mediating the differences in risk are largely unknown. Senescence has been implicated as a causative cellular mechanism for many diseases, including cancer, and may affect the risk for CRC. Senescent fibroblasts that accumulate in tissues secondary to aging and oxidative stress have been shown to promote cancer formation via a senescence‐associated secretory phenotype (SASP). In this study, we assessed the role of senescence and the SASP in CRC formation. Using primary human colon tissue, we found an accumulation of senescent fibroblasts in normal tissues from individuals with advanced adenomas or carcinomas in comparison with individuals with no polyps or CRC. In in vitro and ex vivo model systems, we induced senescence using oxidative stress in colon fibroblasts and demonstrated that the senescent fibroblasts secrete GDF15 as an essential SASP factor that promotes cell proliferation, migration, and invasion in colon adenoma and CRC cell lines as well as primary colon organoids via the MAPK and PI3K signaling pathways. In addition, we observed increased mRNA expression of GDF15 in primary normal colon tissue from people at increased risk for CRC in comparison with average risk individuals. These findings implicate the importance of a senescence‐associated tissue microenvironment and the secretory factor GDF15 in promoting CRC formation.

## 1 INTRODUCTION

The risk of developing colorectal cancer (CRC) varies between people. Epidemiologic factors associated with an increased risk for CRC include old age, red meat consumption, tobacco use, obesity and inactivity, as well as others (Kuipers et al., 2015). Most CRC arise in adenomatous polyps and develop via an adenoma–carcinoma sequence, which results from the accumulation of genetic and epigenetic alterations in colon epithelial cells that drive tumor initiation and progression (Coppede, Lopomo, Spisni, & Migliore, 2014). In addition to cell‐autonomous genetic and epigenetic alterations, other factors related to the tumor microenvironment have also been implicated in CRC, including chronic inflammation (Ryu, Park, Bang, Kang, & Hwangbo, 2016). These cell‐autonomous factors presumably play a role in determining the likelihood that nascent adenomatous polyp cells will progress into adenomas as well as the likelihood of malignant transformation of an adenoma into CRC.

Of note, advanced age and diet are two prominent risk factors for colon adenomas and CRC (Brenner et al., 2007). The molecular and cellular mechanisms that mediate the CRC risk associated with aging and diet are incompletely understood. With regard to diet, it has been proposed that oxidative factors in foods like red meat may promote CRC formation (Montonen et al., 2013). It is also possible that the effects of the diet on the microbiome may influence CRC risk by increasing microbiome mediated oxidative stress in the colon (Gagniere et al., 2016). With regard to aging, a variety of potential mechanisms have been proposed to mediate age‐related disease, including the accumulation of misprocessed protein aggregates, increased oxidative stress injury, DNA damage, and cellular senescence (Chandrasekaran, Idelchik, & Melendez, 2017). Oxidative stress and reactive oxygen species (ROS) accumulate in aging tissue and can induce cellular senescence, which is believed to promote age‐related diseases (Chandrasekaran et al., 2017). In addition, oxidative stress has been associated with an increased risk for colorectal cancer (Mandal, 2017). Interestingly, secreted factors from aged dermal fibroblasts have been shown to modulate ROS (Kaur et al., 2016). Taken together, these observations provided us with a rationale for using an H_2_O_2_‐induced senescence for studying tumor progression.

ROS‐induced senescence is a particularly compelling mechanism that may mediate the variance in CRC risk among people, in light of the evidence showing aging and diet, the two most prominent risk factors for CRC, can promote an oxidative state in the colon (Stone, Krishnan, Campbell, & Palau, 2014). Cellular senescence is an irreversible growth arrest that occurs in response to a variety of different stimuli, including oxidative stress, radiation, and oncogenic stress (Rodier & Campisi, 2011). It has been shown that senescent cells can accumulate in aging organisms secondary to impaired cell clearance by the aging immune system. Senescent cells that chronically accumulate in tissues have been shown to have the potential to create a pro‐tumorigenic microenvironment in some tissues, such as prostate and breast, through the senescence‐associated secretory phenotype (SASP), which is characterized by the increased expression of cytokines, chemokines, growth factors, and proteases (Acosta et al, 2013; Brenner et al., 2007; de Jager et al., 2011; Fray & Dickinson, 2001). Fibroblast cell lines induced to undergo senescence can stimulate malignant and premalignant skin epithelial cells to proliferate in culture and form tumors in mice through SASP‐mediated processes (Krtolica, Parrinello, Lockett, Desprez, & Campisi, 2001). Other studies have shown that the SASP can facilitate skin, lung, and prostate cancer initiation, progression, and metastasis through effects on the extracellular matrix (ECM), through the induction of angiogenic factors, and through inhibiting the anti‐tumor response of lymphocytes (Demaria et al., 2014; Toso et al., 2014; Val et al., 2015).

Given that CRC is an age‐ and diet‐related disease and that the cellular and molecular mechanisms that underlie adenomatous polyp initiation and transformation are only partly understood, we carried out a series of studies to determine whether senescence‐associated mechanisms may play a role in the polyp‐to‐CRC sequence. In this study, we provide both correlative and functional evidence that senescent fibroblasts and an essential SASP factor, GDF15, induce physiological and molecular changes that promote the adenoma–carcinoma initiation and progression sequence in the colon.

## 2 RESULTS

### 2.1 The colon stroma of individuals with advanced adenomas or CRC has an increased number of senescent cells

**Figure 1:**
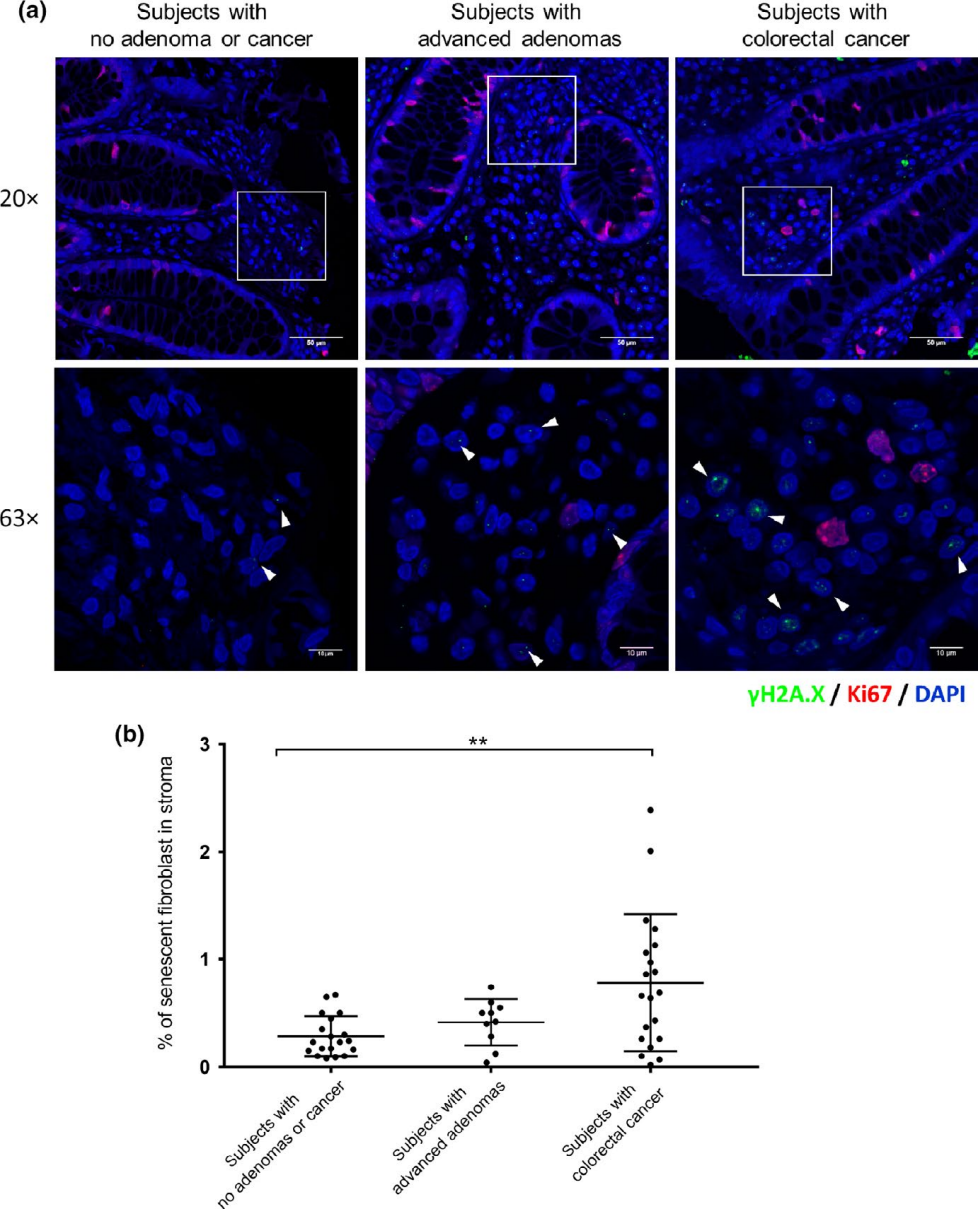
Increased number of senescent fibroblasts in normal colon from individuals with advanced adenomas or CRC. (a) Representative sample of human colon mucosa subjected to immunofluorescence for γ‐H2A.X and Ki67. Senescent cells were identified by being γ‐H2A.X (green) positive and Ki67 (red) negative. Lower panels are magnified images of the boxed regions. Arrows denote the senescent cells. (Scale bar = 50 µm for upper panel, scale bar = 10 µm for lower panel.) (b) Percentage of senescent fibroblasts in stroma of normal colon mucosa from individuals with no adenomas or CRC (*n* = 19, median age = 59), advanced adenomas (*n* = 10, median age = 60), or CRC (*n* = 20, median age = 59). The statistical significance was determined using a Student's *t* test. Statistically significant differences are indicated: *p < 0.05, **p < 0.01, ***p < 0.001

To determine whether there is an association between the proportion of senescent cells in the colon stroma and the occurrence of colon adenomas or CRC, we evaluated the expression of senescent stromal cells in normal colon mucosa from individuals with no colon polyps, with colon adenomas or with CRC (clinical characteristics of patient samples are provided in Table S1). Senescent stromal cells were identified as negative  for Ki67, which suggests the cell is in growth arrest and positive for γ‐H2A.X, which is an indicator of a DNA damage response (DDR) (arrowheads in Figure 1a). This assay has previously been demonstrated to be reliable for detecting senescent cells in situ in primary human tissues (Narita et al., 2003). We found a significantly greater number of senescent cells in the normal colon from individuals with advanced adenomas (0.42%) and carcinomas (0.78%, *p* < 0.01) compared with polyp‐free colons (0.29%) (Figure 1a,b). This observation suggests that an increased accumulation of senescent stromal cells may mediate polyp initiation and/or the adenoma–CRC transition.

### 2.2 Senescent colon fibroblast exhibits pro‐oncogenic effects on colon epithelial cells and human‐derived colon organoids

To assess the potential tumorigenic effects of senescent fibroblasts on colon epithelial cells, CCD‐18Co, a colon fibroblast cell line, was co‐cultured with colorectal adenoma cell lines (LT‐97, AA/C1) and colorectal carcinoma cell lines (Caco‐2, HT‐29) in a two‐chamber well; the two cell types were kept separate to allow an assessment of the effect of secreted proteins from senescent fibroblasts on the colon epithelial cells. To induce senescence, we treated CCD‐18Co cells with hydrogen peroxide, which causes oxidative stress‐induced senescence (Purcell, Kruger, & Tainsky, 2014). The induction of H_2_O_2_‐mediated senescence was identified by measuring senescence‐associated β galactosidase (SA‐β‐gal) activity and the expression of γ‐H2A.X. An optimal H_2_O_2_ treatment (400 µM) for inducing senescence in >80% of the CCD‐18Co cells was determined and used in the subsequent experiments (Figures S1 and S2).

**Figure 2:**
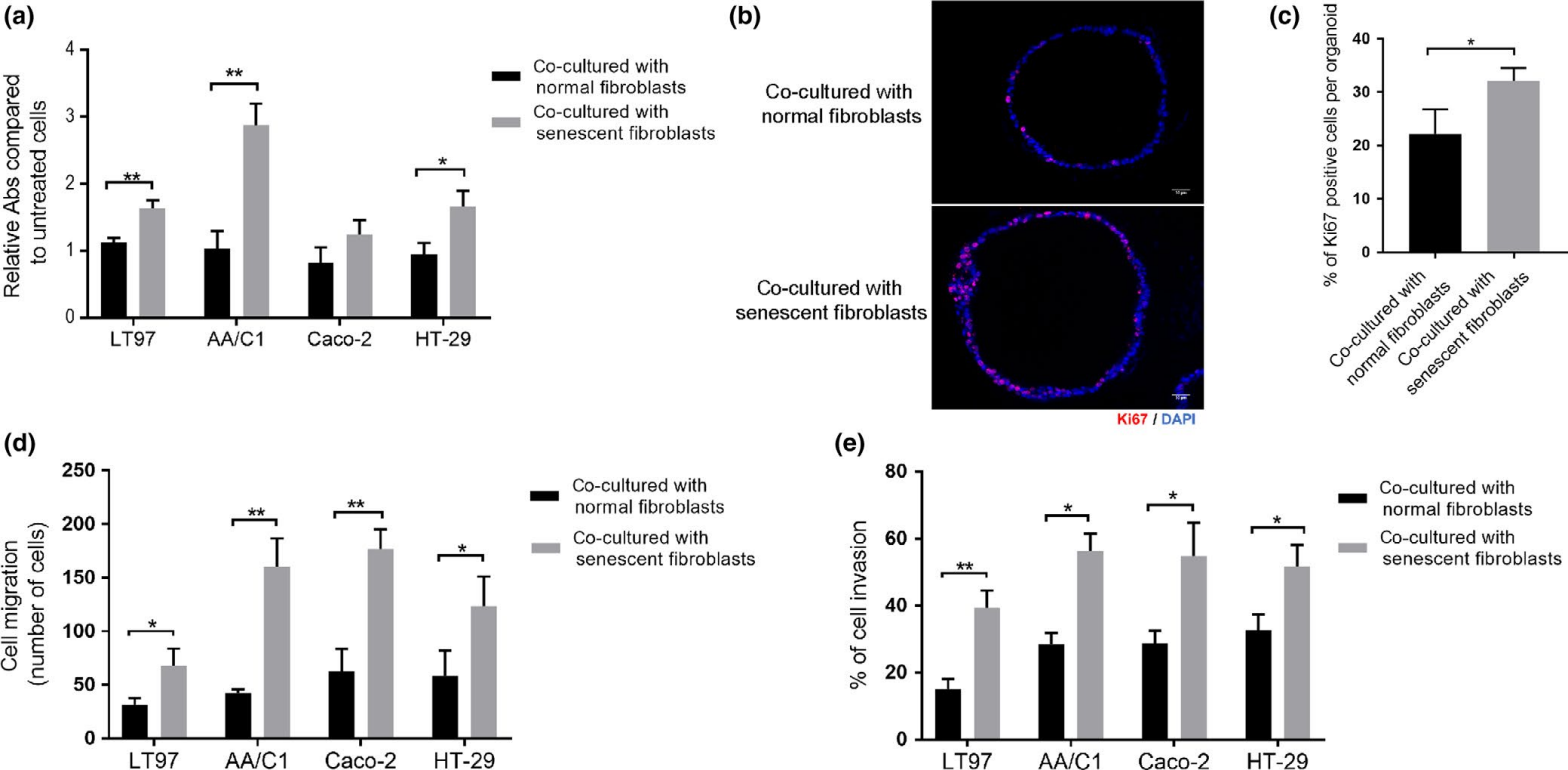
Senescent fibroblasts have oncogenic effects on colon epithelial cell lines and colon organoids. (a) Senescent CCD‐18Co enhance cell proliferation in colorectal adenoma cell lines (LT97 and AA/C1) and CRC cell lines (Caco‐2 and HT‐29) compared with normal CCD‐18Co fibroblasts (*n* = 3 independent experiments). (b, c) Senescent CCD‐18Co promote cell proliferation in human colon organoids. Cell proliferation was measured by immunofluorescence staining for Ki67. Representative images of Ki67 staining in colon organoids (b). Quantification of Ki67‐positive cells in colon organoids co‐cultured with senescent CCD‐18Co vs. normal CCD‐18Co (c). All quantification used TissueQuest software and is based on three independent experiments run on 20 organoids. (d, e) Senescent CCD‐18Co significantly promoted cell migration (d) and cell invasion (e) in the LT97, AA/C1, Caco‐2, and HT‐29 cell lines (*n* = 3 independent experiments). Statistical significance was determined using a Student's *t* test. Statistically significant differences are indicated: *p < 0.05, **p < 0.01, ***p < 0.001

We next assessed the effects of senescent fibroblasts (CCD‐18Co) on colon epithelial cell growth, and we found that senescent fibroblasts significantly increased proliferation in most of the adenoma and CRC cells compared to normal fibroblasts (Figure 2a). The growth‐promoting effect of senescent fibroblasts on epithelial cells was also tested in primary human colon organoids. The organoids were established from normal human colorectal epithelium. As with the adenoma and CRC cell lines, we observed increased proliferation in the organoids co‐cultured with senescent CCD‐18Co when compared to normal CCD‐18Co (Figure 2b,c). Our results indicate that senescent fibroblasts may promote adenoma and/or CRC formation by inducing proliferation in colon epithelial cells through a paracrine‐mediated process.

We next studied the effect of senescent fibroblasts on cell migration and invasion. In comparison with normal fibroblasts, we found the senescent fibroblasts significantly induced cell migration and invasion, as measured by Boyden chamber assays, of the colon adenoma cell lines (LT97 and AA/C1) and CRC cell lines (HT‐29 and Caco‐2) (Figure 2d,e), and by the spheroid matrix invasion assay of HT29 cells (Figure S3).

### 2.3 Identification and validation of senescence‐associated gene expression in colon fibroblasts

After observing senescent fibroblasts have potentially pro‐tumorigenic effects in vitro and that these effects appeared to be mediated through soluble factors, we conducted studies to assess global changes in gene expression patterns to determine whether the senescent fibroblasts might have acquired a senescence‐associated secretory phenotype (SASP).

**Figure 3:**
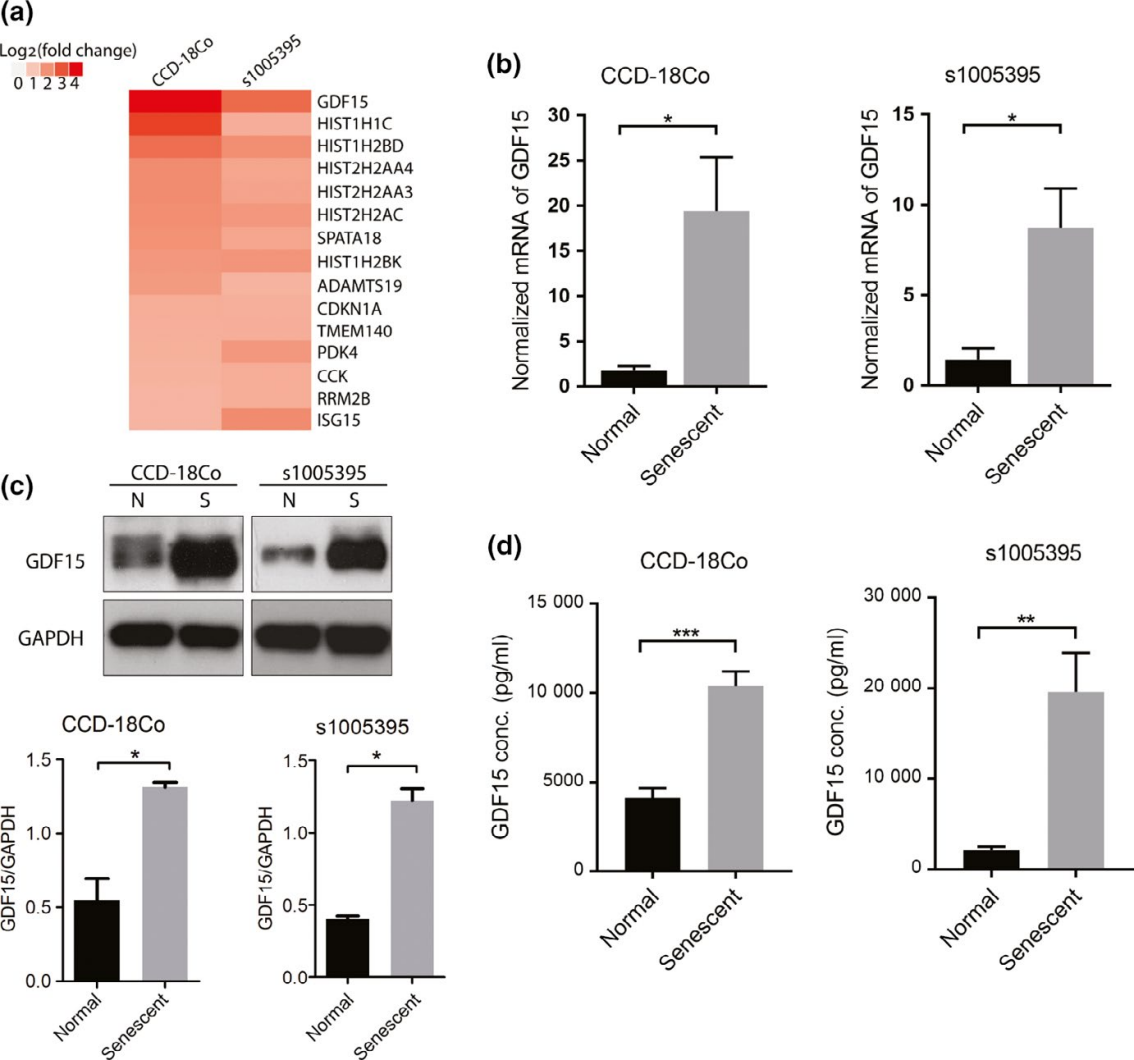
Identification and validation of GDF15 as a component of the SASP in senescent colon fibroblasts. (a) Representative heat map of microarray analysis of senescent fibroblasts. The most highly expressed genes in the senescent colon fibroblast cell line CCD‐18Co (left column) and primary colon fibroblast s1005395 (right column) are shown. GDF15 was up‐regulated in both fibroblast lines after the induction of senescence. (b) The mRNA expression of GDF15 was confirmed to be increased in senescent CCD‐18Co (left panel) and senescent s1005395 (right panel) using RT–PCR (*n* = 3 independent experiments). (c) Western blot analysis of GDF15 expression shows GDF15 was highly induced in senescent fibroblasts compared to normal fibroblast cells in CCD‐18Co (left panel) and s1005395 (right panel) (N=normal, S=senescent). (d) Secreted GDF15 is increased in the conditioned media of the senescent fibroblast cell lines. ELISAs were run on conditioned medium collected from senescent fibroblasts. Increased GDF15 concentrations compared to the control cell lines for both CCD‐18Co (left panel) and s1005395 (right panel) were observed (*n* = 3 independent experiments). The statistical significance was assessed using a Student's *t* test. Statistically significant differences are indicated: *p < 0.05, **p < 0.01, ***p < 0.001

To identify genes differentially expressed in senescent colon fibroblasts, we induced senescence in a primary colon fibroblast line (s1005395) and in CCD‐18Co, and assessed gene expression patterns using Human HT‐12 v4 Expression BeadChip microarrays. The isolated primary colon fibroblast was analyzed by immunofluorescence for vimentin and EpCAM in order to confirm its mesenchymal features (Figure S4). Significant gene expression alterations associated with senescence were identified by comparison with the control fibroblast gene expression patterns, with a 2 fold change and *p* < 0.05 considered significant. Because the SASP consists of proteins overexpressed in senescent cells, we focused on genes that were consistently up‐regulated in the senescent fibroblasts. We identified 102 genes increased in s1005395 and 141 genes increased in CCD‐18Co (see Table S3–S4 for the significantly up‐regulated gene list). GDF15 was among the most highly induced genes in both the primary fibroblast line and the CCD‐18Co cell line (Figure 3a). Because of the potential for GDF15 to mediating the SASP effects we observed in the in vitro studies noted above, we focused our attention on this protein. The increased mRNA and protein expression levels of GDF15 detected by the microarrays were validated using orthogonal assays (Figure 3b,c). In addition, increased secreted GDF15 was also observed in the conditioned media from the senescent fibroblast lines (Figure 3d). We further demonstrated that GDF15 induction is a consistent senescence‐associated effect in fibroblasts by observing increased GDF15 mRNA and protein expression in two other primary fibroblast lines isolated from additional study subjects (s1005379 and s1005405) (Figure S5).

### 2.4 Senescence‐associated GDF15 secretion induces cell proliferation, migration, and invasion in colon epithelial cells

**Figure 4:**
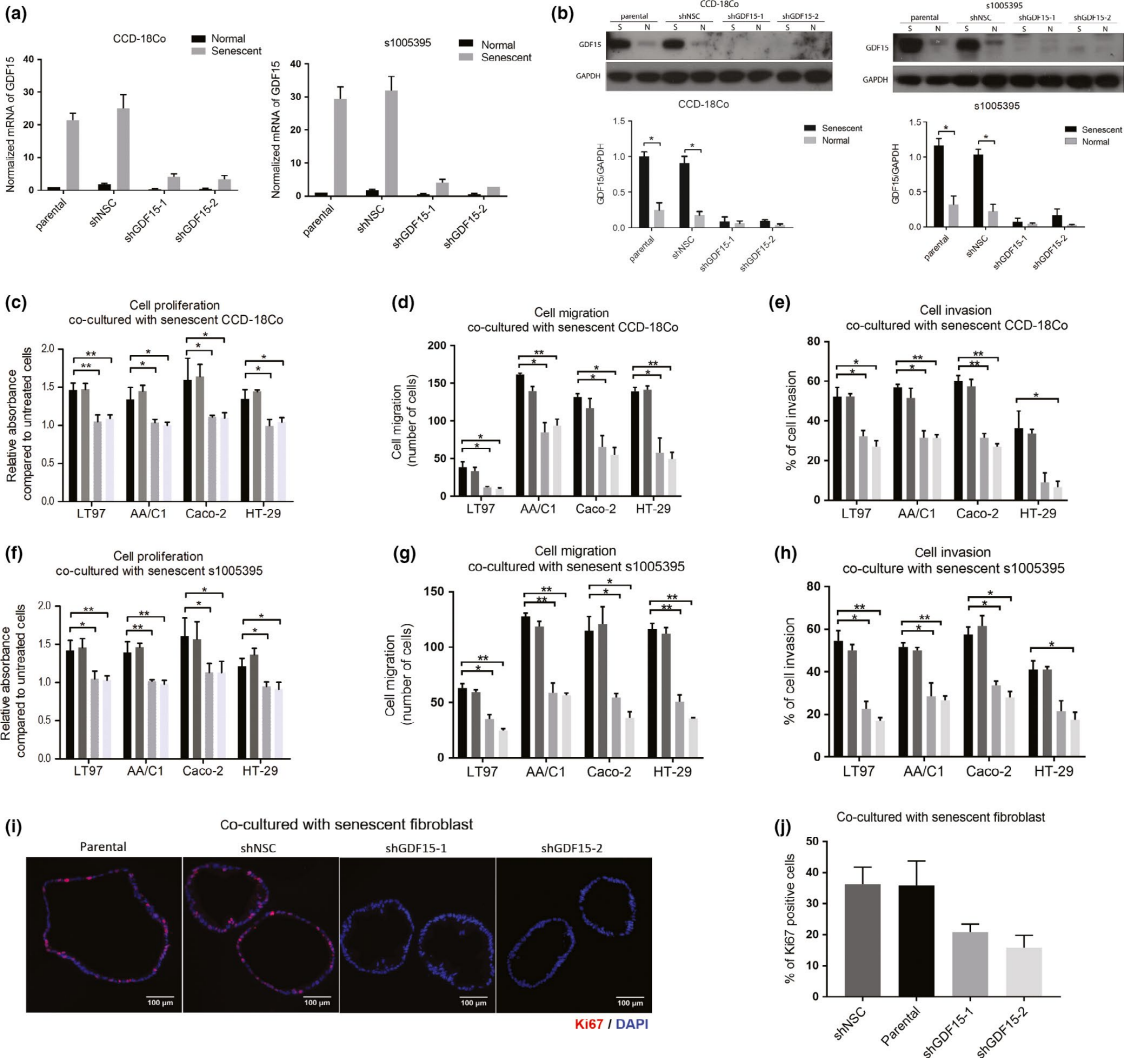
The pro‐oncogenic effects of co‐cultured senescent fibroblasts on colon epithelial cells are abolished by GDF15 knockdown in senescent fibroblast cell lines. (a) The mRNA of GDF15 was reduced in senescent CCD‐18Co (left panel) and s1005395 (right panel) transfected with shGDF15 (shGDF15‐1 and shGDF15‐2) compared to cells transfected with scrambled shRNA and to parental cells (*n* = 2 independent experiments). (b) The protein expression of GDF15 was decreased in senescent CCD‐18Co (left panel) and s1005395 (right panel) transfected with shGDF15 compared to cells transfected with scrambled shRNA and to parental cells (S=senescent, N=normal). (c–e) Cell proliferation (c), cell migration (d), and cell invasion (e) were suppressed in colon epithelial cells (including LT97, AA/C1, Caco‐2, and HT‐29) co‐cultured with senescent CCD‐18Co with shGDF15 knockdown (*n* = 2–3 independent experiments). (f–h) Cell proliferation (f), migration (g), and invasion (h) abilities were reduced in colon epithelial cells (including LT97, AA/C1, Caco‐2, and HT‐29) co‐cultured with senescent primary fibroblast s1005395 with shGDF15 knockdown (*n* = 2–3 independent experiments). (i, j) Cell proliferation of colon organoids was repressed when organoids were co‐cultured with senescent fibroblasts with shGDF15 knockdown. Cell proliferation was measured by immunofluorescent staining of Ki67 (red). Representative images of Ki67 staining in colon organoids (i). Quantification of Ki67‐positive cell in colon organoids (j). All quantification is based on two independent experiments run on 20 organoids and was done using TissueQuest software. The statistical significance was determined using a Student's *t* test. Statistically significant differences are indicated: *p < 0.05, **p < 0.01, ***p < 0.001

In light of our observation that senescent fibroblasts promote cell proliferation, migration, and invasion in colorectal adenoma and CRC cell lines, and the discovery of GDF15 among the most up‐regulated genes in the senescent fibroblasts, we next assessed whether secreted GDF15 from senescent fibroblasts is a mediator of the pro‐tumorigenic effects of the senescent cells on colon epithelial cells. To do this, we suppressed GDF15 expression in the s1005395 and CCD‐18Co lines using shRNA (Figure 4a,b) and then assessed the effects of the senescent fibroblasts with knocked‐down GDF15 on co‐cultured colon epithelial cells. In comparison with co‐cultured colon epithelial cells with senescent fibroblasts with intact GDF15 expression, cell proliferation, migration, and invasion abilities were repressed in both the adenoma and CRC cell lines (Figure 4c–h). In addition, when colon organoids were co‐cultured with CCD‐18Co with knocked‐down GDF15, cell proliferation induced by senescent fibroblasts was reduced in comparison with the organoids co‐cultured with senescent fibroblasts with intact GDF15 expression (Figure4i,j).

### 2.5 Secreted GDF15 from senescent colon fibroblasts mediates pro‐oncogenic effects through the activation of ERK, p38, and AKT

**Figure 5:**
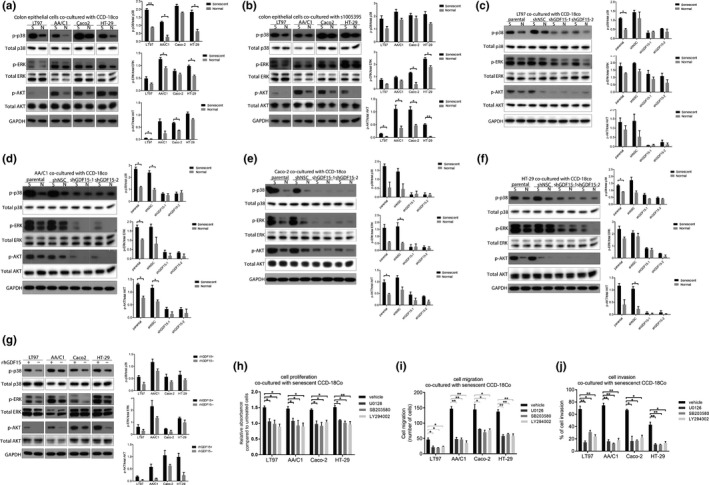
Secreted GDF15 from senescent fibroblasts mediates pro‐oncogenic effects on epithelial cells through activating the MAPK and PI3K pathways. (a, b) The phosphorylation of p38, ERK, and AKT was induced in colon epithelial cells (LT97, AA/C1, Caco‐2, and HT‐29) that were co‐cultured with senescent CCD‐18Co(A) or s1005395(B) cells compared to the epithelial cells grown with normal fibroblasts (S=senescent, N=normal). (c, f) The phosphorylation of p38, ERK, and AKT was reduced in colon epithelial cells, including LT97(C), AA/C1(D), Caco‐2(E), and HT‐29(F) when they were co‐cultured with senescent fibroblast cell line CCD‐18Co after shGDF15 knockdown (S=senescent, N=normal). In all experiments, colon epithelial cell lines and colon fibroblasts were co‐cultured for three days. (g) Human recombinant GDF15 (rhGDF15) induced the phosphorylation of p38, ERK, and AKT in all four colon epithelial cells (LT97, AA/C1, Caco‐2, and HT‐29). Colon epithelial cell lines were treated with rhGDF15 (20 ng/ml) for three days. The medium with rhGDF15 was refreshed every day. DPBS (0.1%) was used as a vehicle control treatment. (h–j) Cell proliferation (h), migration (i), and invasion (j) were reduced in colon epithelial cells (LT97, AA/C1, Caco‐2, and HT‐29) treated with U0126 (ERK inhibitor), SB203580 (p38 inhibitor), and LY294002 (AKT inhibitor). In cell proliferation, migration, and invasion assay, colon epithelial cell lines and senescent fibroblasts were co‐cultured in the presence of inhibitors, including U0126 (5 µM), SB203580 (10 µM), and LY294002 (10 µM). DMSO (0.1%) served as the vehicle control treatment (*n* = 2 independent experiments). The statistical significance was determined using a Student's *t* test. Statistically significant differences are indicated: *p < 0.05, **p < 0.01, ***p < 0.001

In order to further assess the role of secreted GDF15 from senescent colon fibroblasts, we examined the activation status in colon epithelial cells of signaling pathways previously shown to be regulated by GDF15. Recent studies have identified GFRAL as the receptor for GDF15 and have shown that GFRAL activation by GDF15 results in the activation of a number of signaling pathways including the PI3K, ERK, and p38 MAPK pathways (Yang et al., 2017). Thus, we assessed the ERK, AKT, and p38 MAPK signaling pathways in colorectal adenoma and CRC cell lines co‐cultured with senescent fibroblasts. We observed increased phospho‐ERK, phospho‐p38 MAPK, and phospho‐AKT in the majority of the cell lines. The AKT pathway was the most significantly activated pathway in the cell lines. We noted that Caco‐2 was not as strongly induced in the ERK and p38 pathways compared to the other cell lines (Figure 5a,b). This is of interest because we also observed less senescent fibroblast‐induced cell proliferation of Caco‐2 (Figure 2a). To confirm that these signaling pathways were activated by GDF15, we silenced GDF15 expression in the CCD‐18Co cell line and then assessed the levels of phosphorylated ERK, p38, and AKT in the co‐cultured epithelial cell lines. We found significantly reduced levels of p‐ERK and p‐p38 and p‐AKT in LT97, AA/C1, Caco‐2, and HT‐29 cell lines (Figure 5c,f). We also treated the colon epithelial cell lines with recombinant human GDF15 and observed increased ERK phosphorylation and AKT phosphorylation, and to a less extent p38 phosphorylation, after treatment (Figure 5g). (See Figure S6 for data that demonstrate the accuracy of the assays.)

To determine whether the activation of MAPK and PI3K signaling pathways is required for GDF15‐mediated pro‐oncogenic activity, colon epithelial cells were co‐cultured with senescent fibroblasts CCD‐18Co in the presence of pharmacological inhibitors of ERK (U0126), p38 (SB203580), and AKT(LY294002). The promotion of cell proliferation, cell migration, and cell invasion activities that was stimulated by GDF15 was blocked by each of these inhibitors (Figure 5h–j). Of note, we did not observe a significant difference in proliferation of HT29 and LT‐97 cells in response to the inhibitor agents used either individually or in combination for 72 hr (Figure S7). Overall, our results provide evidence that GDF15 is a mediator of colon fibroblast SASP‐induced cell proliferation, migration, and invasion through activation of ERK, p38, and/or PI3K signaling pathways.

### 2.6 GDF15 expression is elevated in the normal colon mucosa of individuals with advanced colon adenomas or CRC

**Figure 6:**
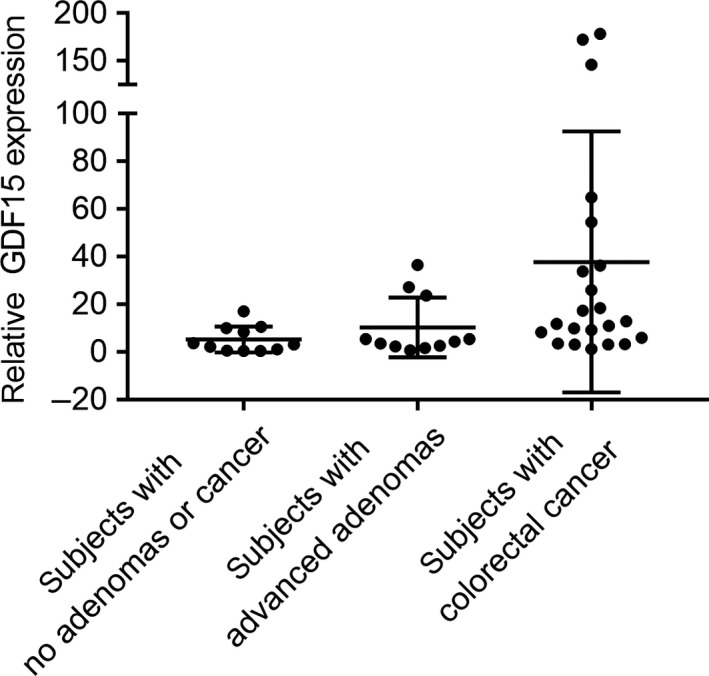
The expression of GDF15 in the colon of patients with no adenomas or CRC, with advanced adenoma(s), and with CRC. As measured by RT–PCR, the mRNA expression of GDF15 was elevated in the normal colon mucosa from subjects with advanced adenoma(s) (*n* = 11) or CRC (*n* = 22) in comparison with subjects with no polyps or CRC (*n* = 11). The clinical characteristics of the study subjects are provided in Table S2 **.** Statistically significant differences are indicated: *p < 0.05, **p < 0.01, ***p < 0.001

In light of our studies showing GDF15 is a mediator of oncogenic effects of the colon SASP in vitro, we next assessed GDF15 expression in primary human colon mucosa of subjects with no adenomas or CRC, with advanced adenomas, and with CRC. We found GDF15 was more highly expressed in subjects with advanced adenomas or CRC compared to subjects with no adenomas or cancer (Figure 6). We also assessed whether age was a potential confounding factor on GDF15 expression. Using tumor tissue or normal colon samples in the TCGA CRC dataset, we found no significant positive correlation between the gene expression level of GDF15 and age (Figure S8). These results are consistent with our in vitro results that GDF15 is a tissue microenvironment‐derived SASP factor that can promote CRC formation.

## 3 DISCUSSION

Our studies provide evidence that the accumulation of senescent cells in the colon is a mechanism that increases the risk for CRC. We observed an increased number of senescent fibroblasts in the normal colon mucosa from patients with advanced adenomas and CRCs compared to healthy polyp‐free individuals. Furthermore, we showed senescent fibroblasts can stimulate cell proliferation, migration, and invasion in epithelial cells via secreted factors. Of note, among the colon epithelial cell lines tested, AA/C1 and HT29 had similar levels of induction of all the biological responses assessed by the senescent fibroblasts, while the resulting increase in proliferation of LT97 and of Caco2 was moderate compared to AA/C1 and HT29. The differential response of these lines to the senescent fibroblasts could reflect the underlying differences in intrinsic characteristics between the colon cancer and adenoma cell lines (i.e., epigenomic and genomic alterations and basal signal pathway activation status). There is considerable known and unknown heterogeneity among the cell lines, including *TP53* mutation status, and one of these differences could be responsible for the differences we observed. We also showed one of the senescence‐associated secretory proteins, GDF15, is sufficient for inducing the oncogenic effects of the colon SASP on epithelial cells and appears to do so by activating the MAPK and PI3K pathways. We found GDF15 expression is elevated in the normal colon from patients with advanced adenomas or CRC. In aggregate, these data provide evidence that the SASP and GDF15 play a role in creating a pro‐oncogenic microenvironment in the colon and may facilitate adenomatous polyp formation and the adenoma‐to‐carcinoma progression sequence.

Advanced age and diet are two of the most significant factors found to associate with an increased risk for CRC (Kuipers et al., 2015). A variety of mechanisms have been proposed that may mediate the increased CRC risk resulting from advanced age and diet. These mechanisms include, among others, an increased accumulation of senescent cells, oxidative stress, inflammaging, and diet‐induced inflammation (Chandrasekaran et al., 2017; Finkel, Serrano, & Blasco, 2007; White et al., 2014). Our data showing elevated senescent cells and GDF15 in the colons of individuals with advanced adenomas or CRC suggest that the accumulation of senescent cells is an in vivo phenomenon in the colon that may mediate increased CRC risk. We propose that the accumulation of the senescent cells may be secondary to increased oxidative stress from a diet rich in red meat, aging, and/or the microbiome (Chandrasekaran et al., 2017; Gagniere et al., 2016). Our in vitro studies used oxidative stress to induce senescence in colon fibroblasts, which we propose is relevant to the in vivo setting.

One of the most fundamental aspects of CRC tumorigenesis is the accumulation of mutations (e.g., mutant *APC, KRAS, BRAF*, and *TP53)* and epigenetic alternations (e.g., aberrant DNA methylation of *MLH1*) that mediate the initiation and formation of CRC (Grady & Pritchard, 2014; Okugawa, Grady, & Goel, 2015). Our studies now suggest that CRC formation is also affected by the tissue microenvironment and that the accumulation of senescent cells may mediate CRC risk via the secretion of SASP factors. We identified GDF15 as a major SASP factor in the colon of people with concurrent advanced colon neoplasms and showed it is secreted by senescent colon fibroblasts. The SASP is a dynamic, complex secretion profile associated with cellular senescence and varies in different cell types. It includes cytokines, chemokines, growth factors, and extracellular proteases that can affect surrounding cells (Coppe, Desprez, Krtolica, & Campisi, 2010). For instance, MMPs and IL6 secreted by senescent fibroblasts have been shown to contribute to a tumor‐promoting microenvironment in the breast and prostate and tumor progression (Liu & Hornsby, 2007; Tsai, Chuang, Little, & Yuan, 2005). Our study systematically investigated the ability of GDF15 to induce hallmark behaviors of cancer in a variety of human colon adenoma and CRC cell lines and human colon organoids (Hanahan & Weinberg, 2011). Based on our findings, we propose that GDF15 is a prominent SASP element secreted from senescent colorectal fibroblasts that creates a chronic inflammation microenvironment in the colon mucosa that predisposes people to CRC.

GDF15 (growth differentiation factor 15), also known as MIC‐1 (macrophage inhibitory cytokine‐1), belongs to the TGF‐β superfamily (Bootcov et al., 1997). Previous studies have provided evidence that GDF15 is involved in a variety of pathological conditions, including cardiovascular disease, diabetes, and some types of cancer (Breit, Tsai, & Brown, 2017; Coppede et al., 2014). Elevated serum levels of GDF15 and increased GDF15 tissue expression in patients with metastatic prostate, pancreatic, breast, and colorectal cancer have also been observed (Bansal et al., 2017; Lee et al., 2003; Wang et al., 2014; Welsh et al., 2003). Although these studies have suggested that increased GDF15 expression may mediate aggressive cancer cell behaviors, little is known about its potential to induce cancer transformation of normal epithelial cells (Bansal et al., 2017; Lee et al., 2003; Wang et al., 2014; Welsh et al., 2003). Li et al. (2016) reported that GDF15 promotes CRC cell metastasis through activating EMT process by activating the TGF‐β receptor and SMAD2/3 pathway. In our study, we did not observe a significant change in the activation of SMAD2 and/or SMAD3 when the colon epithelial cells were co‐cultured with senescent fibroblasts (Figure S8). Instead, we found that senescence‐associated GDF15 mediated tumor‐promoting effects through the MAPK and PI3K pathways. Our results are consistent with the possibility that GDF15 is mediating its effects via GFRAL, a recently discovered GDF15 receptor that can activate ERK1/2, p38, and AKT (Yang et al., 2017). GFRAL and its co‐receptor RET are expressed in the colon, which supports this possibility. (See https://www.proteinatlas.org/ENSG00000187871-GFRAL/tissue/colon.)

In our study, the use of primary colon fibroblasts and colon organoids isolated from human colon grown in three‐dimensional culture systems created a realistic ex vivo system that is the most relevant system currently available for the study of human disease processes, albeit with its own limitations (Crespo et al., 2017; Fatehullah, Tan, & Barker, 2016). In the future, a more sophisticated co‐culture system that includes the microbiome, stromal fibroblasts, and immune cells would be useful for the study of cellular senescence and the SASP. Our results also suggest that therapeutic agents that inhibit the ERK/MAPK, AKT, and p38 pathways, which are being used in preclinical studies or are approved for use in the clinic, may have a use in the clinic via their effects on GDF15 signaling. They have shown variable clinical activity against colorectal cancer, and some of them (e.g., MAPK inhibitors) appear to be more effective when used in combination with other therapies (Ahronian et al., 2015). Thus, for those agents that have benign side effect profiles, there might be potential that they could be used in combination with a GDF15 inhibitor for the prevention or treatment of colorectal cancer in the future (Baek et al., 2009; Bauskin et al., 2006; Roth et al., 2010). Furthermore, in light of the promising preclinical results for the combination of BRAF inhibitors with MAPK inhibitors in CRC (Ahronian et al., 2015; Ursem, Atreya, & Van Loon, 2018), it is compelling to consider the use of GDF15 inhibitors, such as AV‐380 (Aveo) with anti‐BRAF therapies in the future (Of note, the HT29 cell line carries mutant BRAF V600E.). Further studies are needed to determine the potential for this approach to be used for reducing the risk of CRC.

Overall, our studies provide evidence that the accumulation of senescent fibroblasts in the normal colon increases CRC risk, through the generation of SASP and increased GDF15, and suggest new approaches for CRC prevention.

## 4 EXPERIMENTAL PROCEDURES

### 4.1 Cell lines and human tissue samples

All studies in this manuscript were reviewed and approved by the Cancer Consortium (Fred Hutchinson Cancer Research Center, University of Washington School of Medicine, Seattle Cancer Care Alliance) IRB committee.

The HEK293 cell line, colon fibroblast cell line CCD‐18Co, and CRC cell lines Caco‐2 and HT‐29 were purchased from American Type Culture Collection (ATCC, Manassas, VA, USA). The AA/C1 cell line was kindly provided by C. Paraskeva's (University of Bristol, Bristol, UK). The LT97 was a generous gift from M. Richter (University of Vienna, Austria). Cell culture conditions are described in Supporting Methods. Forty‐nine formalin‐fixed paraffin‐embedded (FFPE) and 44 fresh frozen (FF) normal colon tissues were from polyp‐free patients, patients with advanced adenomas, and patients with CRC. For the isolation of primary colon fibroblasts and colon organoids, three fresh normal colon tissues were obtained from patients with CRC. The normal colon used in these studies was located at least 10 cm from the tumor or polyp.

### 4.2 Isolation and cell culture of human colon organoids

The procedure of colon organoid culture was carried out as described by Sato et. al (Sato et al., 2011). Detailed methods are provided in Supporting Information.

### 4.3 Isolation of primary human colon fibroblasts

The procedure of colon organoid culture was carried out as described by Yoo et al (Khalil, Nie, Edwards, & Yoo, 2013). Detailed procedures are provided in Supporting Methods.

### 4.4 Cellular senescence induction 

The protocol was modified from the method described previously (Chen, Ozanne, & Hales, 2007) and provided in Supporting Methods.

### 4.5 Insert co‐culture experiment

All co‐culture experiments were performed using permeable supports with microporous membranes which allow two different cell populations growing in the same culture vessel. The co‐culture system was used in cell proliferation, migration, and invasion assays for colon epithelial cell lines and colon organoids. Detailed methods are described in each assay.

### 4.6 Microarray analysis

Total RNA was isolated from fibroblasts using TRIzol Reagent (Thermo Fisher Scientific). RNA concentrations were measured at 260 nm using NanoDrop (Thermo Fisher Scientific). RNA was subsequently labeled and hybridized to Human HT‐12 v4 Expression BeadChip microarrays (Illumina). Bioinformatics analysis was performed using Bioconductor package “lumi” and “limma” in R version 1.0.136. The microarray data are available upon request.

### 4.7 GDF15 knockdown

Detailed methods are provided in Supporting Information.

### 4.8 Immunofluorescence

Detailed methods are provided in Supporting Information.

### 4.9 RNA extraction from cell lines and primary tissues

Detailed methods are provided in Supporting Information.

### 4.10 Quantitative real‐time PCR

mRNAs level of GDF15 was determined by a SYBR assay. Detailed methods and primer sequences are provided in Supporting Information.

### 4.11 Western blotting

Western blotting experiments were conducted following routine protocols. Detailed methods including reagents and antibodies are provided in Supplemental Methods.

### 4.12 ELISA

ELISA of GDF15 was performed using GDF15 human ELISA kit (Thermo Fisher Scientific) according to the manufacturer's protocol.

### 4.13 Colon epithelial cells–fibroblasts co‐culture

The colon epithelial cells–fibroblasts co‐culture was performed using the Corning^®^ HTS Transwell^®^ (Corning). The detailed protocol is provided in Supplemental Methods.

### 4.14 Cell proliferation assay

Cell proliferation assay was performed using CellTiter 97^®^ Aq_ueous_ Non‐Radioactive Cell Proliferation Assay (MTS) (Promega, cat# G5421) according to the manufacturer's protocol. Optical density was measured at 490 nm using a plate reader (Molecular Devices).

### 4.15 Cell migration assay

Cell motility was measured on FluoroBlok Inserts with 8 µm pores (Corning, cat# 351152). Briefly, certain number of colon epithelial cells (3 × 10^5^ cells/ insert for LT‐97 and AA/C1, 5 × 10^5^ cells/ insert for Caco‐2 and HT‐29) were plated onto inserts. For co‐culture experiment, 2 × 10^4^ fibroblasts were plated onto 24‐well plate. After 24 hr, inserts were removed and stained with DAPI (4′,6‐diamidino‐2‐phenylindole). Membrane filters were imaged on a Nikon Elapse E300 Microscope using a 20× objective. Three representative fields were counted from each experimental group.

### 4.16 Cell invasion assay

The cell invasion assay was performed according to the protocol developed by Corning https://www.corning.com/worldwide/en/products/life-sciences/keymatch/transwell-assay-protocol.html and provided in Supporting Methods.

### 4.17 Spheroid invasion assay

Spheroid invasion assay was modified from methods previously described in Poudel et al., (2018), and details are provided in Supporting Methods.

### 4.18 Correlation of GDF15 expression with patient age in the TCGA‐COAD data

The correlation analysis was performed using RNA‐seq datasets from 40 normal and 271 primary colon cancer tissue (COAD) samples obtained from the TCGA (https://tcga-data.nci.nih.gov/tcga). The detailed analysis is provided in Supplemental Methods. Original RNA expression values (normalized read counts) were used for the downstream analyses. The normalized RNA‐Seq (By Illumina HiSeq platform) counts of all genes from the legacy database were aligned against the hg19 reference genome and clinical information of samples using the *TCGAbiolinks* R package. After data cleaning, 40 normal and 271 primary colon cancer tissue (COAD) samples with their tumor stage and age annotations remained and were used for the relevant studies.

### 4.19 Statistical analyses

The microarray data analyses applied R/Bioconductor statistical software. R/Bioc libraries used included lumi and limma.

GraphPad Prism 7 software (GraphPad) was used to analyze all data to determine statistical significance. Data were expressed as the mean ± *SEM*. Student's *t* test or Mann–Whitney *U* test was used to compare differences between groups in all experiments. *p* < 0.05 was considered as significant.

## CONFLICT OF INTEREST

None declared.
