# Supplementary material for: Senescence‐associated tissue microenvironment promotes colon cancer formation through the secretory factor GDF15
Source: Aging Cell. 2019 Aug 6;18(6):e13013. doi: 10.1111/acel.13013 (PMC6826139; doi:10.1111/acel.13013)
Supplement: Supplementary file 1 [file ACEL-18-e13013-s001.docx]

**
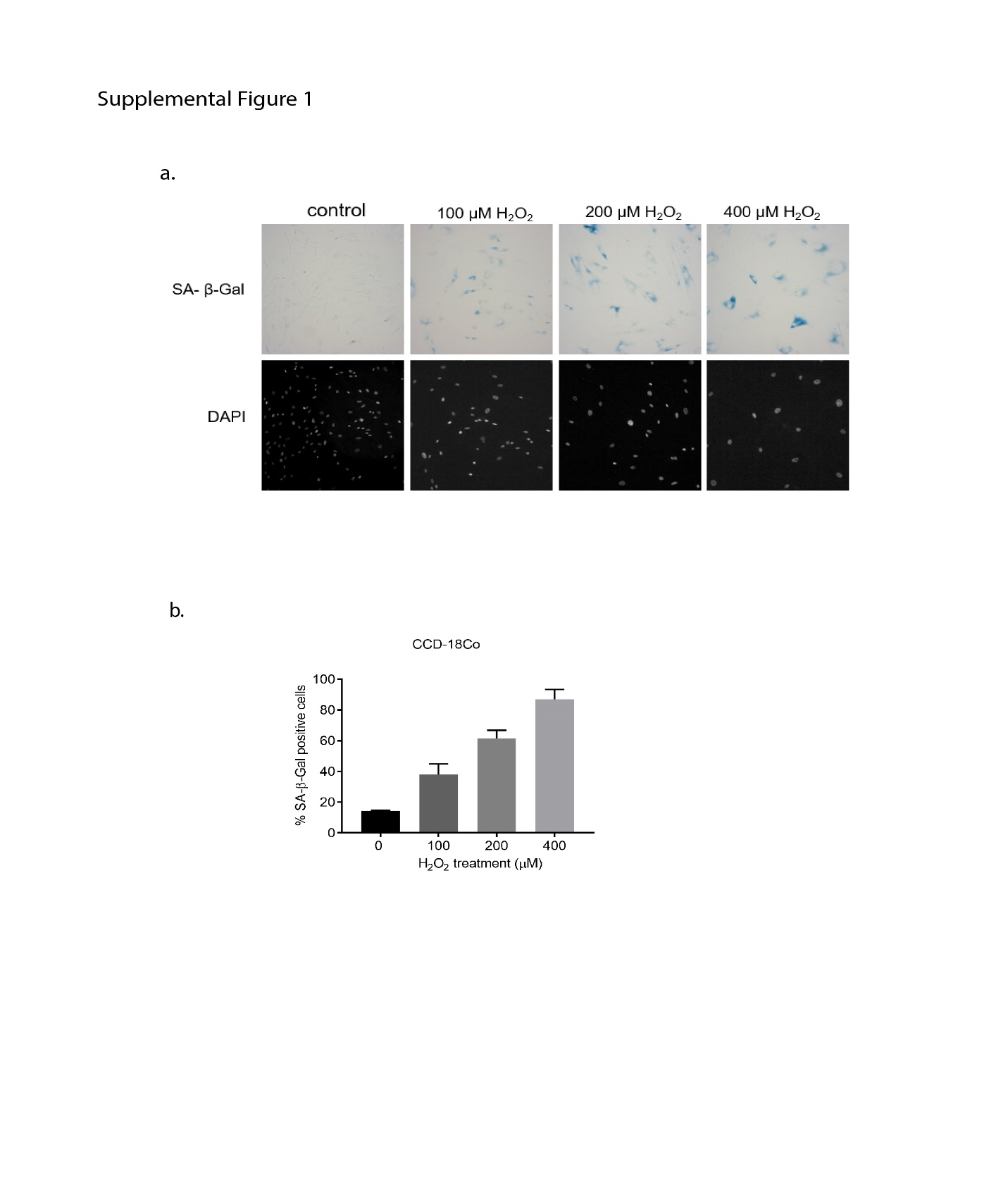
Supplemental Figure S1**

**Supplemental Figure S1. Assessment of hydrogen peroxide induced senescence in CCD-18Co cells using SA-β-gal staining after CCD-18Co was exposed to different concentrations of H_2_O_2_.** (A) Representative images of SA-β-gal staining in CCD-18Co. (B) Quantification of SA-β-gal staining in CCD-18Co.


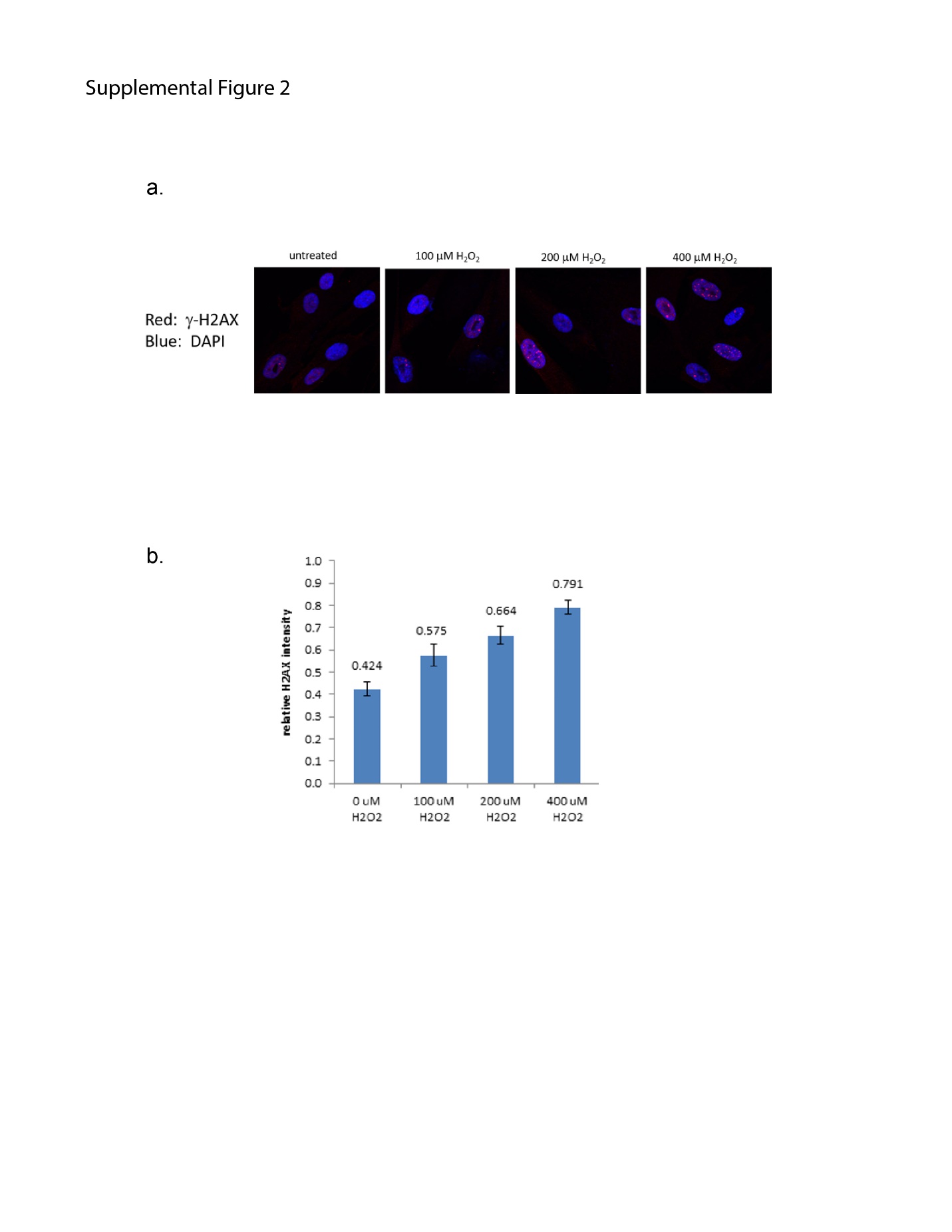
**Supplemental Figure S2**

**Supplemental Figure S2. Indirect assessment of senescence associated chromatin foci (SACF) induction with escalating doses of hydrogen peroxide using immunofluorescence for γH2A.X in CCD-18Co cells.** (A) Representative images of γH2A.X in CCD-18Co. (B) Quantification of γH2A.X in CCD-18Co.


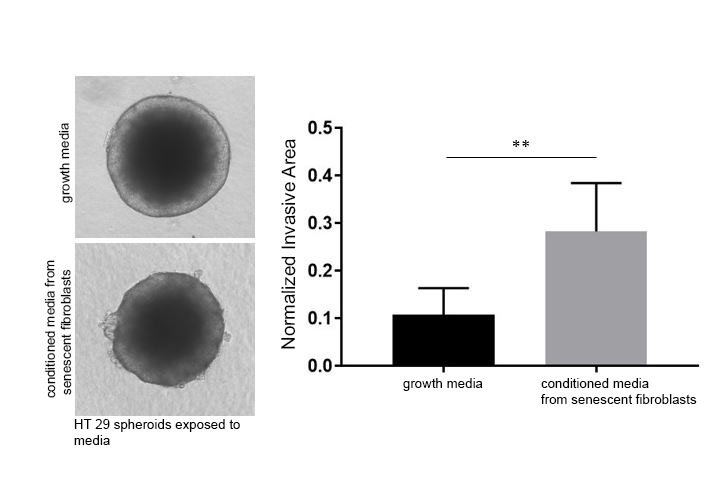
**Supplemental Figure S3**

**Supplemental Figure S3. Invasion of colon cancer cell line HT29 spheroids treated with conditioned media from senescent CCD18-Co or normal growth media.** Invasion of HT29 spheroids (original magnification x20) exposed to condition media from senescent CCD18-Co cells (N=7), compared to ones exposed to control media (N=8) treated for one week (Mann-Whitney two-tailed, P=0.0047).


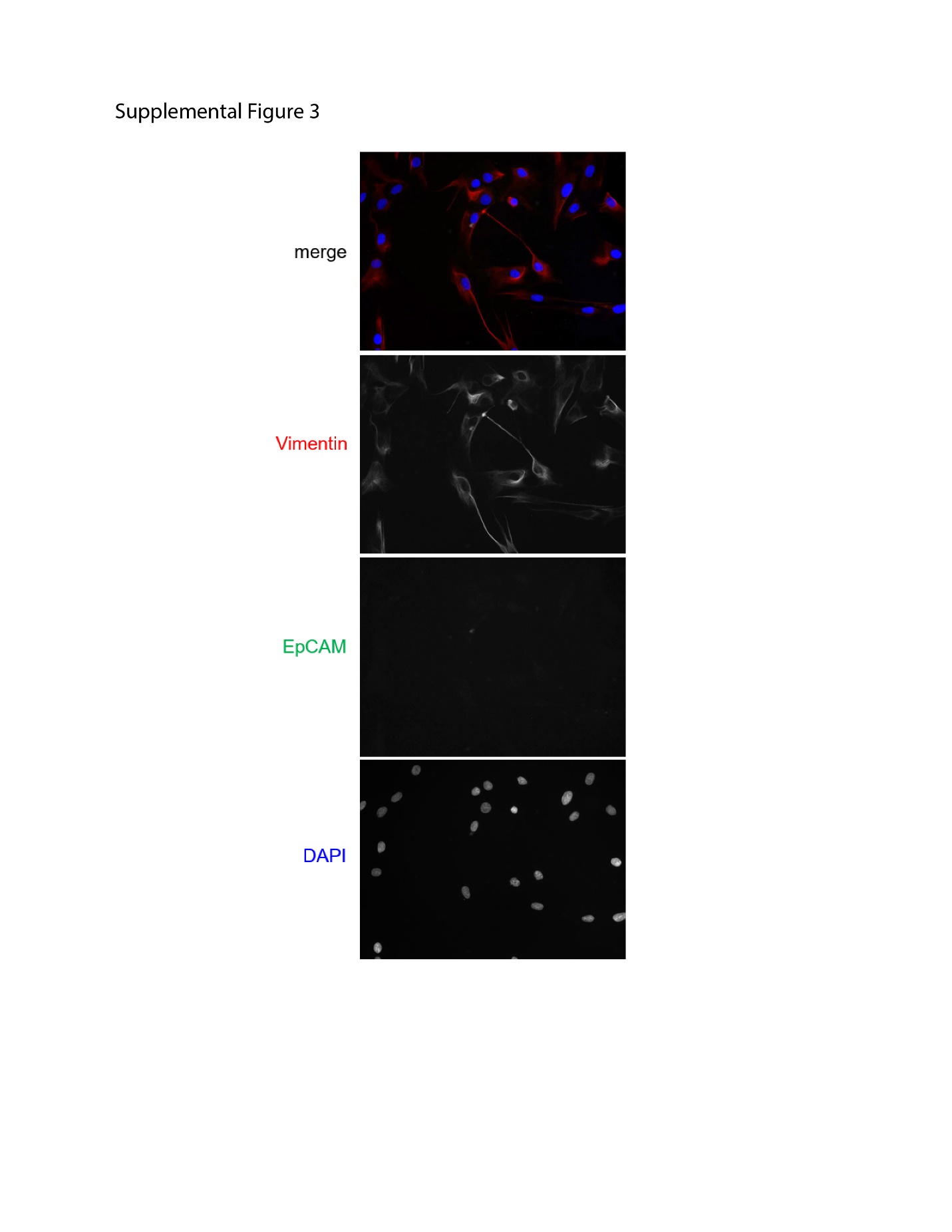
**Supplemental Figure S4**

**Supplemental Figure S4. Cell characterization of isolated primary colon fibroblast cells by immunofluorescence.** Primary colon fibroblast cells were identified by being vimentin (red) positive and EpCAM (green) negative.

**Supplemental Figure S5**


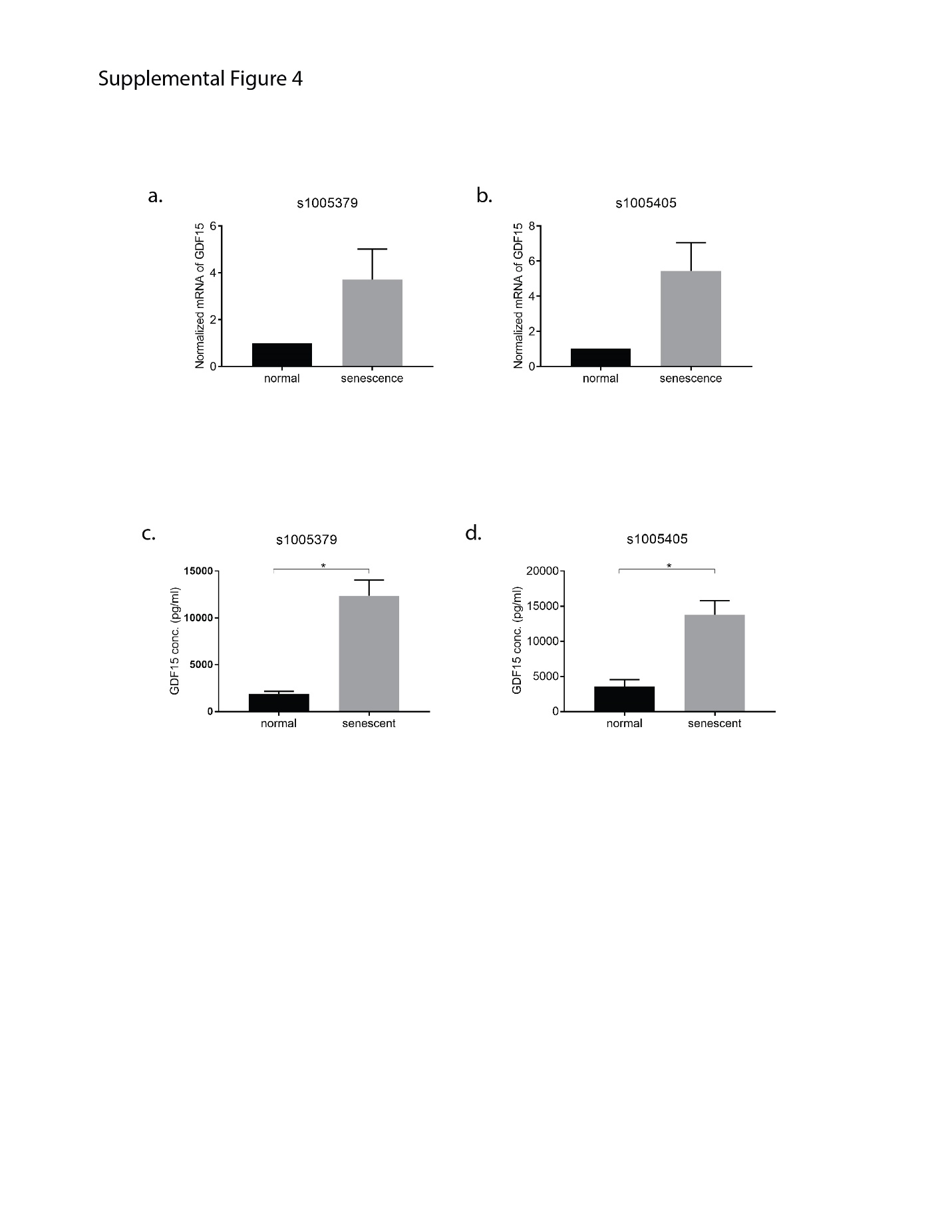


**Supplemental Figure S5. GDF15 expression is increased in senescent primary colon fibroblast lines s1005379 and s1005405.** (A-B) mRNA expression of GDF15 was increased after the induction of senescence of two independent colon fibroblast cell lines s1005379 and s1005405. (C-D) The conditioned medium collected from senescent fibroblast lines s1005379 and s1005405 shows increased GDF15 expression.

**Supplemental Figure S6**


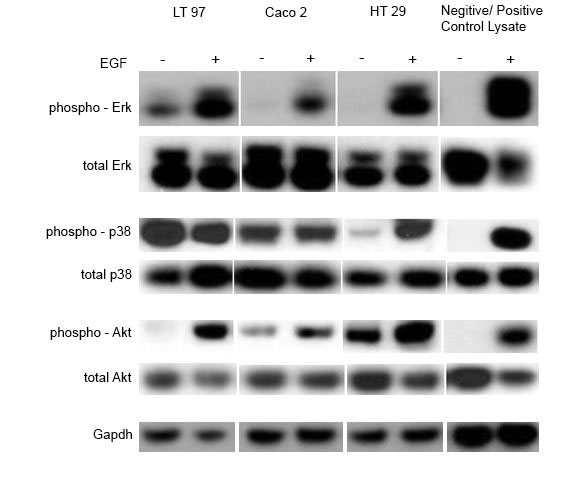


**Supplemental Figure S6.** The phosphorylation of Erk, p38 and Akt was measured in cells at baseline and after treatment with EGF. Negative and positive control cell lysates from Cell Signaling Technologies are included. GAPDH was used as a loading control.

**Supplemental Figure S7**


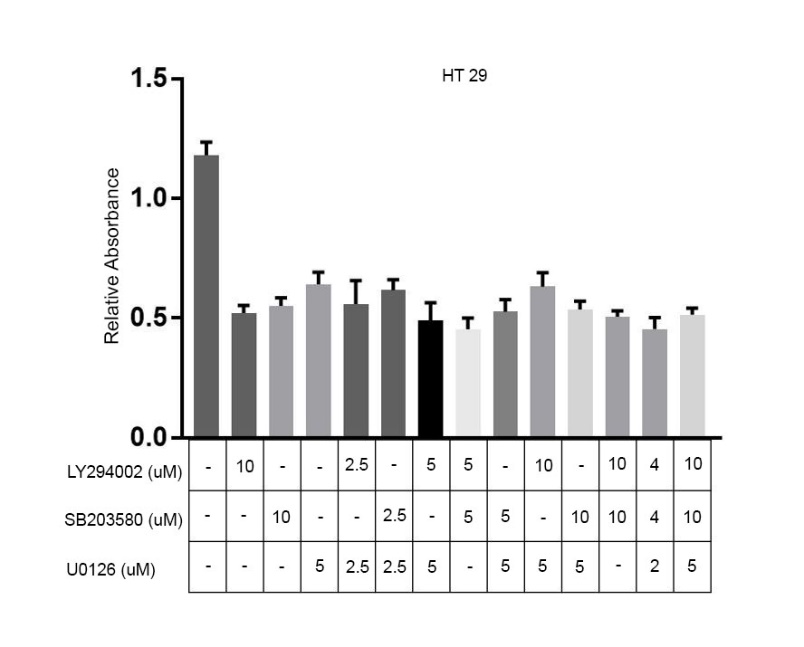


**a.**

**


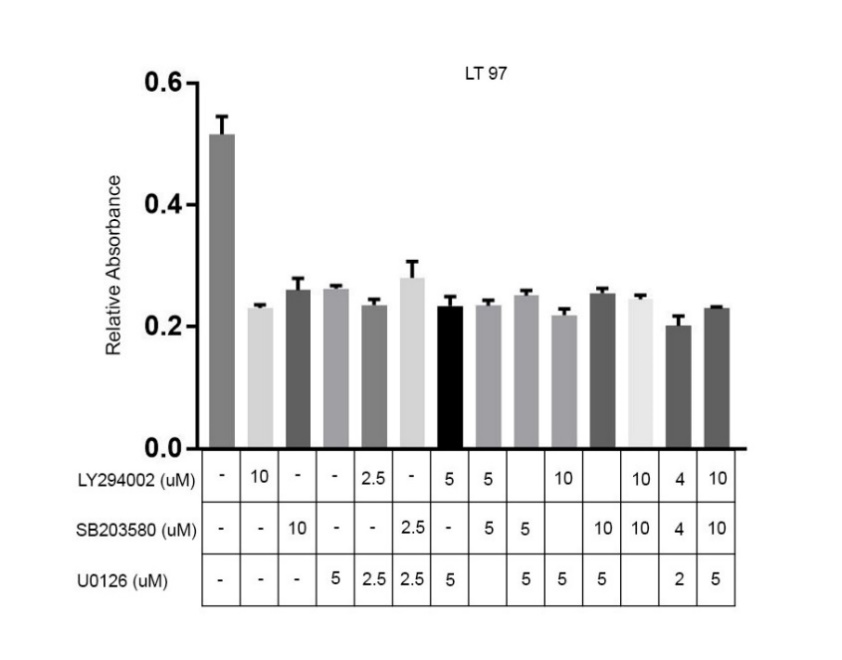


**LT 97**

****

**

**

**b.**

**Supplemental Figure S7.** Proliferation assay of a representative CRC cell line, HT 29, and representative colorectal adenoma cell line, LT 97, co-cultured with senescent CCD18 cells and exposed to various concentrations of inhibitors U0126 (2, 2.5, and 5uM), SB203580 (4, 5, and 10uM), and LY294002 (4, 5, and 10uM) for 72 hours. ** indicates the significant difference seen in proliferation, measured by relative absorbance, in cells treated with inhibitors vs. vehicle (Student t test p < 0.01).


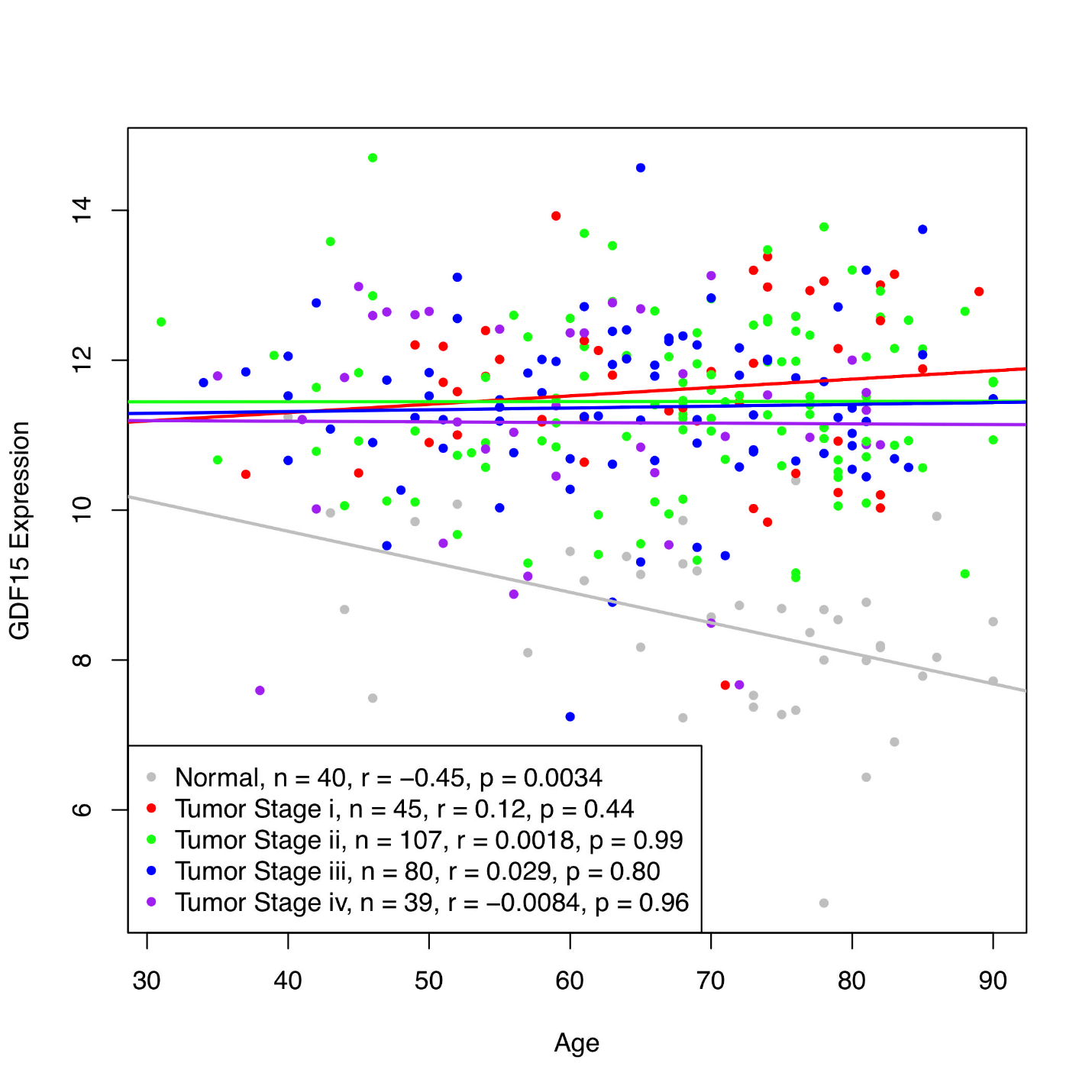
**Supplemental Figure S8**

**Supplemental Figure S8. Correlation of GDF15 expression with patient age in TCGA-COAD data for normal and tumor (COAD) samples**. Different colors represent different tumor stages. Sample sizes (n), correlations (r) and P-values (p) are shown in the figure.


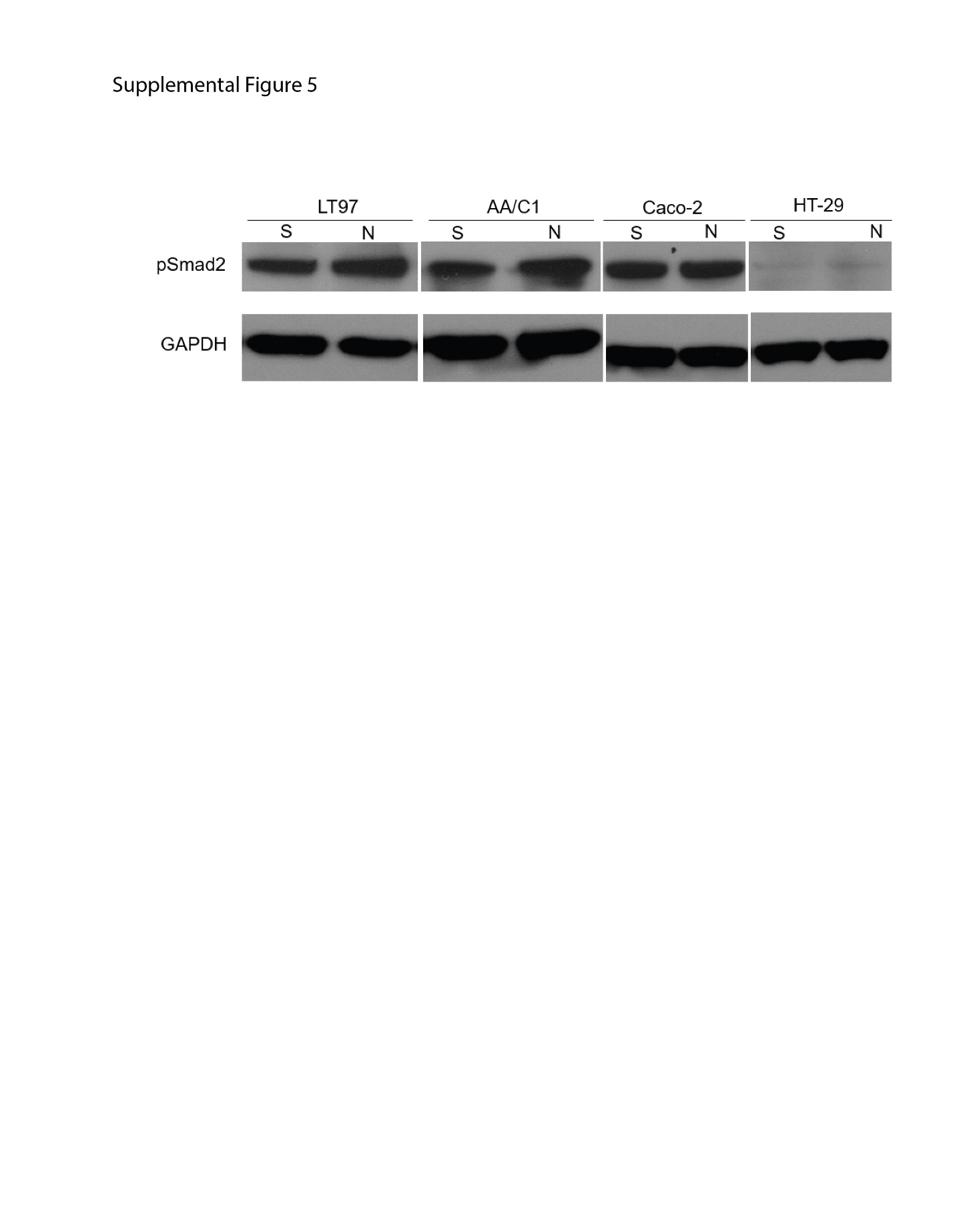
**Supplemental Figure S9**

**Supplemental Figure S9.** The expression of phospho-Smad2 in colon epithelial cells (LT97, AA/C1, Caco-2 and HT-29) co-cultured with senescent CCD-18Co compared to the epithelial cells grown with normal fibroblasts (S – senescent, N – normal).
