# Supplementary material for: Senescence‐associated tissue microenvironment promotes colon cancer formation through the secretory factor GDF15
Source: Aging Cell. 2019 Aug 6;18(6):e13013. doi: 10.1111/acel.13013 (PMC6826139; doi:10.1111/acel.13013)
Supplement: Supplementary file 2 [file ACEL-18-e13013-s002.docx]

| **Supplemental Table 1. subject information for the samples used for the assessment of senescent cells** | | | | |
| --- | --- | --- | --- | --- |
|  | | **subjects with no adenomas or cancer** | **subjects with advanced adenomas** | **subjects with colorectal cancer** |
| **Age groups** | 21-30 | 2 | 0 | 0 |
|  | 31-40 | 1 | 1 | 0 |
|  | 41-50 | 3 | 0 | 3 |
|  | 51-60 | 5 | 5 | 8 |
|  | 61-70 | 8 | 2 | 8 |
|  | 71-80 | 0 | 2 | 1 |
| **Median of age** | | 59.0 | 60.0 | 59.0 |
| **p value, reference subjects set: no adenomas(s) or CRC** | |  | NS | NS |
| **total** | | 19 | 10 | 20 |

| **Supplemental Table 2. Patient information for the GDF15 RT-qPCR analysis** | | | | |
| --- | --- | --- | --- | --- |
|  | | **Subjects with no adenomas or cancer** | **Subjects with advanced adenomas** | **Subjects with colorectal cancer** |
| **Age groups** | 31-40 | 2 | 2 | 1 |
|  | 41-50 | 2 | 1 | 3 |
|  | 51-60 | 4 | 3 | 11 |
|  | 61-70 | 2 | 2 | 4 |
|  | 71-80 | 1 | 2 | 1 |
|  | 81-90 |  | 1 | 2 |
| **Median of age** | | 58 | 60 | 57.5 |
| **p value, reference subject set: no adenomas(s) or CRC** | |  | NS | NS |
| **total** | | 11 | 11 | 22 |

**Supplemental Table 3**. A list of a subset of the top genes significantly upregulated in the senescent CCD18-Co fibroblasts treated with 400 uM H_2_O_2_, compared to non-senescent control fibroblast cells (log (fold change)>1, Adj. p < 0.05). The list is ordered by logFC values, *GDF15* is in bold

| **TargetID** | **logFC** | **AveExpr** | **P.Value** | **adj.P.Val** |
| --- | --- | --- | --- | --- |
| MMP3 | 5.327928 | 10.0646 | 4.2792E-09 | 3.20783E-06 |
| **GDF15** | **3.696559** | **11.10342** | **1.47446E-09** | **1.65796E-06** |
| MMP1 | 3.535321 | 9.343756 | 1.61849E-07 | 2.53521E-05 |
| HIST1H1C | 2.841583 | 10.9718 | 1.72067E-09 | 1.84268E-06 |
| ASNS | 2.7055 | 10.85053 | 4.5371E-10 | 1.27543E-06 |
| PSAT1 | 2.356902 | 10.48842 | 1.36101E-10 | 6.88374E-07 |
| TRIB3 | 2.356628 | 9.777724 | 1.56936E-07 | 2.50959E-05 |
| PLIN2 | 2.268926 | 11.8785 | 2.2211E-07 | 3.04424E-05 |
| PLIN2 | 2.223855 | 10.29897 | 2.05736E-06 | 0.000115381 |
| HIST1H2BD | 2.173468 | 8.76201 | 2.59194E-09 | 2.33949E-06 |
| IL13RA2 | 2.031596 | 8.809171 | 3.35568E-07 | 3.72807E-05 |
| ASNS | 1.95615 | 9.551632 | 6.41067E-09 | 3.70321E-06 |
| DDIT3 | 1.913997 | 8.749927 | 2.79412E-05 | 0.000657991 |
| CCND2 | 1.910796 | 10.11086 | 2.9736E-08 | 1.02662E-05 |
| ANGPTL4 | 1.84225 | 10.96566 | 7.40229E-08 | 1.57053E-05 |
| ULBP1 | 1.78131 | 8.737068 | 6.79727E-10 | 1.47054E-06 |
| HIST2H2AC | 1.780547 | 10.84506 | 6.57129E-08 | 1.54677E-05 |
| ATF5 | 1.76402 | 9.769665 | 6.42204E-09 | 3.70321E-06 |
| HIST1H4H | 1.755017 | 7.666174 | 5.33933E-07 | 5.04522E-05 |
| HIST2H2AA4 | 1.739292 | 10.97415 | 4.18816E-07 | 4.33637E-05 |
| HIST2H2AA3 | 1.736722 | 11.53988 | 6.98952E-08 | 1.57053E-05 |
| CLDN1 | 1.731557 | 8.202972 | 1.47088E-05 | 0.000431272 |
| PCNA | 1.725865 | 9.247973 | 3.25222E-07 | 3.69872E-05 |
| HIST1H2AC | 1.717587 | 8.833888 | 3.81664E-08 | 1.18089E-05 |
| HIST2H2AA3 | 1.674007 | 11.06049 | 1.89353E-07 | 2.80156E-05 |
| SPATA18 | 1.651265 | 8.193773 | 2.98011E-09 | 2.48221E-06 |
| HIST1H2BD | 1.636844 | 7.927127 | 3.76726E-07 | 3.99632E-05 |
| IL24 | 1.556356 | 8.488585 | 0.00093504 | 0.007894225 |
| HIST1H2BK | 1.549425 | 9.327284 | 2.71855E-06 | 0.00013708 |
| SLC3A2 | 1.537943 | 9.504363 | 4.60191E-07 | 4.65609E-05 |
| CCND2 | 1.530356 | 8.583153 | 5.66771E-09 | 3.64175E-06 |
| ADAMTS19 | 1.501866 | 8.117204 | 2.1983E-07 | 3.04424E-05 |
| ISCU | 1.469252 | 10.70812 | 2.41686E-08 | 9.37116E-06 |
| PNPO | 1.469066 | 9.581441 | 3.04381E-07 | 3.63108E-05 |
| SEC11C | 1.466159 | 9.070771 | 4.59687E-08 | 1.26663E-05 |
| LOC100133328 | 1.464869 | 9.305424 | 1.02671E-07 | 1.90823E-05 |
| CMBL | 1.463617 | 9.17016 | 1.06086E-07 | 1.9505E-05 |
| LOC644033 | 1.456276 | 7.852837 | 1.62333E-07 | 2.53521E-05 |
| LOC729779 | 1.444806 | 8.035028 | 1.28306E-06 | 8.53687E-05 |
| PMAIP1 | 1.437191 | 9.084903 | 1.25302E-08 | 5.94627E-06 |
| HIST1H2BC | 1.435662 | 8.049928 | 3.14414E-08 | 1.05535E-05 |
| GBA | 1.433227 | 8.83536 | 1.16357E-07 | 2.04435E-05 |
| HIST1H2BK | 1.423586 | 12.26699 | 1.19363E-07 | 2.0809E-05 |
| PCK2 | 1.412838 | 8.385352 | 4.0998E-06 | 0.000176968 |
| STOM | 1.404425 | 9.839372 | 2.77246E-06 | 0.000139485 |
| FGF2 | 1.399236 | 9.339411 | 2.07003E-06 | 0.000115516 |
| IDH1 | 1.391853 | 9.771718 | 1.32447E-07 | 2.23955E-05 |
| BSCL2 | 1.388958 | 9.690105 | 5.32205E-07 | 5.04522E-05 |
| PPP1R15A | 1.386145 | 10.33206 | 5.39506E-08 | 1.36325E-05 |
| TRNP1 | 1.374351 | 11.20828 | 2.57501E-06 | 0.000131912 |
| ODC1 | 1.360757 | 9.709251 | 7.11318E-07 | 6.12905E-05 |
| NUPR1 | 1.349661 | 9.584884 | 0.000140381 | 0.001965773 |
| SERPINB7 | 1.329088 | 7.897629 | 8.32639E-08 | 1.70229E-05 |
| OBFC2A | 1.324799 | 10.29289 | 1.27181E-06 | 8.52741E-05 |
| SDSL | 1.323106 | 8.158236 | 1.95375E-07 | 2.85311E-05 |
| HSPA9 | 1.316516 | 10.27499 | 3.73162E-07 | 3.99621E-05 |
| LYPLAL1 | 1.308514 | 8.47226 | 3.28125E-07 | 3.69872E-05 |
| GPX1 | 1.293728 | 12.1407 | 1.19296E-06 | 8.27209E-05 |
| LYPLAL1 | 1.291056 | 8.871683 | 2.47968E-06 | 0.00012915 |
| SNHG9 | 1.283401 | 7.860437 | 9.9837E-08 | 1.88675E-05 |
| C20ORF108 | 1.275019 | 9.027026 | 4.34757E-08 | 1.25349E-05 |
| GADD45A | 1.268588 | 9.593172 | 7.69312E-06 | 0.000273129 |
| TP53I3 | 1.267886 | 8.111126 | 1.22356E-06 | 8.38925E-05 |
| OGFRL1 | 1.266188 | 8.390437 | 1.25256E-06 | 8.4846E-05 |
| CPSF4 | 1.265288 | 10.38597 | 4.46846E-06 | 0.000188186 |
| SRXN1 | 1.265224 | 9.217392 | 3.25994E-05 | 0.000728281 |
| KYNU | 1.260205 | 8.759525 | 1.09073E-06 | 7.81848E-05 |
| BEX2 | 1.260158 | 7.42611 | 1.83033E-08 | 7.62263E-06 |
| H2AFZ | 1.241986 | 12.37998 | 1.13743E-07 | 2.01415E-05 |
| CLPTM1 | 1.237913 | 8.081592 | 3.30253E-07 | 3.69872E-05 |
| BCL2L1 | 1.236177 | 8.883041 | 2.13179E-06 | 0.000117793 |
| MARS | 1.226706 | 9.352915 | 7.24639E-06 | 0.000261574 |
| HINT2 | 1.221512 | 8.62552 | 4.58551E-06 | 0.000189914 |
| C15ORF24 | 1.217276 | 11.72731 | 4.42239E-07 | 4.52069E-05 |
| LARP6 | 1.216957 | 8.959004 | 2.04215E-06 | 0.000115102 |
| WARS | 1.215248 | 9.797883 | 1.49678E-06 | 9.28498E-05 |
| H2AFJ | 1.211625 | 11.51491 | 1.44126E-06 | 9.02693E-05 |
| WARS | 1.211141 | 10.39253 | 4.83615E-08 | 1.30208E-05 |
| TSPAN10 | 1.209803 | 8.451577 | 5.13019E-08 | 1.34155E-05 |
| HIST1H2BE | 1.196503 | 7.616146 | 1.11565E-07 | 2.01415E-05 |
| GBA | 1.196337 | 8.340585 | 3.39697E-08 | 1.11457E-05 |
| HAX1 | 1.195759 | 9.461618 | 3.45969E-05 | 0.000759815 |
| GAMT | 1.193086 | 9.410378 | 5.28712E-06 | 0.000210446 |
| APH1B | 1.191976 | 8.61737 | 2.44619E-07 | 3.23221E-05 |
| MTHFD2 | 1.190459 | 9.520081 | 2.54617E-06 | 0.000131032 |
| CDKN1A | 1.189306 | 13.71898 | 1.43452E-08 | 6.08697E-06 |
| SQSTM1 | 1.187471 | 13.01844 | 6.19153E-07 | 5.5303E-05 |
| ISG20L1 | 1.185049 | 9.314712 | 3.1983E-06 | 0.000153139 |
| ABCC4 | 1.175215 | 10.06221 | 1.40078E-05 | 0.000414502 |
| LOC440957 | 1.174836 | 8.269549 | 1.19544E-06 | 8.27209E-05 |
| PLAU | 1.173273 | 12.48448 | 1.9436E-05 | 0.000523468 |
| STOM | 1.1681 | 13.04183 | 3.9794E-08 | 1.18089E-05 |
| HS.25318 | 1.165791 | 8.691291 | 3.02772E-05 | 0.000696853 |
| SLC7A11 | 1.165049 | 7.490608 | 2.93259E-08 | 1.02662E-05 |
| P8 | 1.163344 | 8.901301 | 0.000124663 | 0.001811081 |
| C18ORF56 | 1.163175 | 8.264314 | 1.15903E-06 | 8.14244E-05 |
| SERPINE2 | 1.162961 | 13.07823 | 1.44766E-07 | 2.35917E-05 |
| IFRD1 | 1.161423 | 8.078366 | 7.93206E-07 | 6.61448E-05 |
| C7ORF55 | 1.160874 | 8.380762 | 8.08274E-07 | 6.61722E-05 |
| LOC346887 | 1.158052 | 7.933309 | 1.73131E-05 | 0.000486086 |
| ID1 | 1.1538 | 9.233022 | 1.80383E-06 | 0.000106753 |
| CTSL1 | 1.152038 | 11.65538 | 1.33511E-05 | 0.000400337 |
| TMEM140 | 1.143083 | 7.875419 | 5.24626E-06 | 0.00020919 |
| C12ORF5 | 1.142828 | 8.061925 | 4.2143E-07 | 4.33637E-05 |
| DDRGK1 | 1.138409 | 8.788987 | 2.55553E-07 | 3.26542E-05 |
| SNHG1 | 1.136813 | 8.477925 | 9.38908E-06 | 0.000312816 |
| MTHFD2 | 1.136586 | 9.280084 | 6.35962E-06 | 0.000239969 |
| TP53INP1 | 1.134443 | 8.839831 | 8.94253E-08 | 1.76397E-05 |
| SAT1 | 1.133618 | 11.67768 | 1.30599E-06 | 8.58783E-05 |
| C20ORF24 | 1.13027 | 11.57763 | 1.0668E-07 | 1.9505E-05 |
| RPS24 | 1.127841 | 10.07412 | 0.000147349 | 0.002024268 |
| C7ORF10 | 1.126548 | 8.051905 | 8.05E-08 | 1.66953E-05 |
| ABCC4 | 1.12236 | 10.19535 | 3.52073E-06 | 0.000161587 |
| PDK4 | 1.119835 | 7.335136 | 1.00505E-05 | 0.000326625 |
| GADD45A | 1.118813 | 10.31566 | 2.80927E-07 | 3.49048E-05 |
| KYNU | 1.10713 | 8.19919 | 1.44035E-07 | 2.35917E-05 |
| CTSL1 | 1.105128 | 11.92726 | 1.83007E-07 | 2.72559E-05 |
| DHRS7 | 1.101853 | 11.03886 | 7.75087E-06 | 0.000274071 |
| CCK | 1.092152 | 8.773012 | 9.66121E-06 | 0.000318445 |
| C20ORF24 | 1.089732 | 11.59154 | 5.48505E-07 | 5.13972E-05 |
| GOT1 | 1.085144 | 9.076528 | 3.22634E-06 | 0.000153139 |
| GLIPR1 | 1.079751 | 9.543017 | 5.58988E-08 | 1.38946E-05 |
| HIST2H2BE | 1.078742 | 8.608219 | 2.38635E-07 | 3.17671E-05 |
| ISCU | 1.078623 | 8.756015 | 9.43664E-06 | 0.000313936 |
| TOMM34 | 1.076606 | 10.9568 | 3.2452E-06 | 0.000153139 |
| CCDC90B | 1.075224 | 10.74788 | 7.21857E-08 | 1.57053E-05 |
| NARS | 1.060931 | 11.11583 | 1.38159E-07 | 2.30152E-05 |
| LY96 | 1.057197 | 10.41082 | 1.58256E-07 | 2.50959E-05 |
| C20ORF24 | 1.047048 | 10.09418 | 4.31262E-05 | 0.000873295 |
| RPL34 | 1.04375 | 8.209921 | 2.21566E-05 | 0.000563027 |
| ATP6V0B | 1.043032 | 10.14901 | 4.58485E-06 | 0.000189914 |
| RNF167 | 1.041745 | 8.460711 | 1.25853E-06 | 8.49942E-05 |
| GHITM | 1.041441 | 11.76189 | 8.6028E-08 | 1.7274E-05 |
| LOC642489 | 1.037117 | 10.86945 | 1.33805E-07 | 2.24562E-05 |
| PHLDA3 | 1.036609 | 8.374719 | 5.68084E-06 | 0.000221896 |
| BEXL1 | 1.036439 | 10.02755 | 1.95717E-05 | 0.000523986 |
| ATF3 | 1.034848 | 8.123349 | 6.90203E-05 | 0.001199534 |
| RPP40 | 1.034772 | 8.927048 | 6.51596E-05 | 0.001151118 |
| LOC645166 | 1.034708 | 10.16054 | 4.18886E-06 | 0.000179777 |
| RRM2B | 1.033337 | 7.728087 | 6.19697E-07 | 5.5303E-05 |
| LOC729887 | 1.032916 | 7.833793 | 7.97067E-07 | 6.61448E-05 |
| ATP6V0E1 | 1.031735 | 10.72179 | 1.7311E-05 | 0.000486086 |
| RPL29 | 1.031416 | 8.220229 | 1.92901E-05 | 0.000521837 |
| MT1X | 1.027813 | 10.32581 | 0.000398248 | 0.004202816 |
| ISG15 | 1.027079 | 9.571818 | 2.38208E-05 | 0.000589984 |
| PIGY | 1.022918 | 9.877959 | 2.5676E-05 | 0.0006229 |
| TCTA | 1.020483 | 7.594174 | 7.66257E-07 | 6.50278E-05 |
| PSMG1 | 1.017882 | 8.512986 | 2.1927E-05 | 0.000559088 |
| IDH1 | 1.016301 | 7.902703 | 5.98038E-05 | 0.001085494 |
| RPAIN | 1.014553 | 9.908862 | 1.11609E-05 | 0.00035203 |
| CES2 | 1.012926 | 9.108642 | 8.3995E-05 | 0.001366834 |
| NME1 | 1.012347 | 11.23704 | 8.12428E-05 | 0.001340477 |
| PHLDA1 | 1.009315 | 10.21281 | 1.98144E-07 | 2.87488E-05 |
| HSPA4 | 1.007881 | 10.53099 | 4.24845E-06 | 0.000181641 |
| LAGE3 | 1.006288 | 8.719959 | 2.92653E-06 | 0.000145286 |
| MGST2 | 1.001743 | 8.446779 | 1.33414E-06 | 8.64653E-05 |
| MAPKAPK3 | 1.001607 | 9.474959 | 5.79943E-07 | 5.34522E-05 |
| ESYT1 | 1.000962 | 9.695862 | 1.26978E-06 | 8.52741E-05 |

**Supplemental Table 4**. The top genes significantly upregulated (in the senescent primary fibroblasts S1005395 treated with 400 uM H_2_O_2_, compared to non-senescent control fibroblast cells (log (fold change)>1, Adj. p < 0.05). The list is ordered by logFC, *GDF15* is in bold.

| **TargetID** | **logFC** | **AveExpr** | **P.Value** | **adj.P.Val** |
| --- | --- | --- | --- | --- |
| CST1 | 3.133799 | 9.80276 | 1.19133E-12 | 1.37046E-09 |
| IFI6 | 2.663918 | 10.6092 | 7.71702E-10 | 1.94161E-07 |
| PAPPA | 2.552026 | 9.62088 | 1.02664E-13 | 4.11776E-10 |
| **GDF15** | **2.237953** | **9.82177** | **4.14021E-13** | **6.16355E-10** |
| OAS2 | 2.232978 | 9.51331 | 2.31694E-07 | 1.35484E-05 |
| MX1 | 2.132833 | 11.6298 | 4.99267E-08 | 4.2259E-06 |
| IFI44L | 2.04079 | 8.27958 | 8.639E-07 | 3.75019E-05 |
| IFIT3 | 1.97878 | 9.45414 | 7.6343E-07 | 3.41962E-05 |
| MIR1974 | 1.842827 | 8.09865 | 0.000402098 | 0.00394813 |
| ISG15 | 1.755546 | 11.7852 | 1.32689E-06 | 5.20634E-05 |
| FAM43A | 1.694585 | 10.614 | 1.52491E-09 | 3.21014E-07 |
| HIST1H2BD | 1.693824 | 8.43387 | 1.58779E-09 | 3.24772E-07 |
| HIST1H2BK | 1.690898 | 10.9771 | 1.71637E-10 | 6.48329E-08 |
| IL8 | 1.662319 | 12.1217 | 5.66355E-11 | 2.86666E-08 |
| BMP2 | 1.658774 | 8.71276 | 1.20518E-08 | 1.43871E-06 |
| IFIT3 | 1.64619 | 9.19322 | 2.58811E-06 | 8.83486E-05 |
| HERC5 | 1.641994 | 8.09216 | 7.89742E-08 | 6.01452E-06 |
| IFITM1 | 1.637565 | 11.5706 | 7.13151E-09 | 1.01396E-06 |
| TCF21 | 1.626373 | 11.0425 | 7.61609E-09 | 1.05905E-06 |
| HIST1H2BK | 1.610594 | 9.26591 | 5.08673E-10 | 1.39929E-07 |
| PARM1 | 1.609046 | 11.5226 | 1.35093E-11 | 9.58401E-09 |
| LOC728946 | 1.594413 | 8.7493 | 3.84217E-10 | 1.11767E-07 |
| STC1 | 1.583738 | 11.1132 | 6.50055E-06 | 0.000174831 |
| IFI44 | 1.574363 | 8.80222 | 2.68932E-06 | 9.05071E-05 |
| HES4 | 1.565108 | 9.25069 | 1.98777E-11 | 1.25767E-08 |
| IL8 | 1.564084 | 11.1303 | 3.05526E-09 | 5.26003E-07 |
| PDK4 | 1.549836 | 9.17141 | 4.84309E-07 | 2.36163E-05 |
| OAS1 | 1.549509 | 8.25027 | 6.24E-07 | 2.90726E-05 |
| IFIT1 | 1.547254 | 10.6628 | 4.97274E-05 | 0.000825247 |
| HIST1H2AC | 1.544622 | 8.52285 | 7.59044E-09 | 1.05905E-06 |
| SLC40A1 | 1.505296 | 9.20582 | 4.64435E-11 | 2.5552E-08 |
| TCF21 | 1.48833 | 11.8621 | 1.04589E-09 | 2.42839E-07 |
| SNCA | 1.487044 | 8.98617 | 8.27945E-08 | 6.1993E-06 |
| HERC6 | 1.478657 | 8.6455 | 8.7814E-06 | 0.000223581 |
| KCNJ2 | 1.477364 | 8.96021 | 5.54517E-10 | 1.49295E-07 |
| IFIT2 | 1.458568 | 9.36945 | 0.000168122 | 0.002076095 |
| IFI27 | 1.453362 | 12.3453 | 3.04742E-08 | 2.93248E-06 |
| KRTAP1-5 | 1.436277 | 8.79342 | 1.4733E-09 | 3.15984E-07 |
| MX2 | 1.419314 | 9.0043 | 8.81435E-08 | 6.4659E-06 |
| BST2 | 1.414332 | 10.3743 | 8.3252E-08 | 6.20062E-06 |
| ASAM | 1.412824 | 9.77952 | 6.64353E-06 | 0.000178108 |
| PCYOX1 | 1.411654 | 9.45705 | 1.87589E-07 | 1.15511E-05 |
| TNFRSF21 | 1.410321 | 10.1657 | 1.80125E-08 | 1.9509E-06 |
| ANGPT1 | 1.396694 | 9.92162 | 2.36445E-09 | 4.21405E-07 |
| CYP26B1 | 1.393876 | 9.36998 | 2.80065E-10 | 9.45052E-08 |
| SEZ6L2 | 1.360424 | 8.99299 | 3.05333E-10 | 9.63535E-08 |
| HIST2H2AA3 | 1.359055 | 9.37185 | 8.05337E-08 | 6.08402E-06 |
| LOC387763 | 1.34389 | 11.2202 | 1.81153E-08 | 1.9509E-06 |
| SPATA18 | 1.340087 | 9.09846 | 2.2304E-10 | 7.97816E-08 |
| HIST2H2AA4 | 1.338858 | 9.15701 | 1.13623E-09 | 2.59061E-07 |
| PLAT | 1.324611 | 13.1537 | 2.17725E-09 | 4.0516E-07 |
| IRF7 | 1.308248 | 8.72129 | 8.22591E-07 | 3.62055E-05 |
| NPAS1 | 1.299333 | 8.25154 | 2.55024E-10 | 8.84129E-08 |
| HIST1H2BD | 1.287226 | 8.00281 | 1.20007E-09 | 2.68772E-07 |
| PTGS2 | 1.277444 | 12.3778 | 4.06383E-09 | 6.60578E-07 |
| SCG5 | 1.266028 | 10.1046 | 2.88287E-10 | 9.58907E-08 |
| LOC730525 | 1.265837 | 8.97309 | 2.91749E-10 | 9.58907E-08 |
| CALD1 | 1.265331 | 9.07108 | 2.02763E-08 | 2.13814E-06 |
| TIMP3 | 1.260461 | 11.9839 | 3.85623E-07 | 2.00173E-05 |
| LIF | 1.234087 | 8.4676 | 1.2326E-08 | 1.45769E-06 |
| PCYOX1 | 1.233818 | 9.51258 | 3.52438E-08 | 3.23171E-06 |
| LOC100129681 | 1.232593 | 10.1768 | 6.27274E-07 | 2.91285E-05 |
| NFKBIZ | 1.230916 | 10.0817 | 1.56409E-08 | 1.77507E-06 |
| LOC88523 | 1.226158 | 8.93297 | 1.04793E-06 | 4.30534E-05 |
| SGIP1 | 1.202743 | 9.04901 | 7.51827E-09 | 1.05707E-06 |
| MYH11 | 1.201295 | 10.4269 | 8.09108E-09 | 1.10091E-06 |
| OAS1 | 1.198017 | 8.16879 | 7.5633E-06 | 0.00019856 |
| CCK | 1.191171 | 7.74032 | 2.99309E-08 | 2.90226E-06 |
| RRM2B | 1.187373 | 8.79942 | 1.59127E-09 | 3.24772E-07 |
| SPRY1 | 1.17693 | 10.0433 | 1.343E-08 | 1.55503E-06 |
| FAM65C | 1.161613 | 8.40081 | 7.02133E-08 | 5.48444E-06 |
| LEPROT | 1.159628 | 10.9549 | 7.12486E-07 | 3.22836E-05 |
| TMEM140 | 1.157858 | 8.82234 | 8.19828E-09 | 1.10824E-06 |
| HIST1H1C | 1.157572 | 9.7924 | 5.4562E-09 | 8.02823E-07 |
| GPX3 | 1.152651 | 10.7649 | 3.87314E-08 | 3.45146E-06 |
| XAF1 | 1.149986 | 9.59988 | 3.42975E-06 | 0.000107919 |
| OAS1 | 1.14165 | 8.22104 | 1.14894E-06 | 4.65985E-05 |
| CDKN1A | 1.137276 | 13.8571 | 2.25205E-09 | 4.13006E-07 |
| SEMA6A | 1.132832 | 9.19349 | 7.67643E-08 | 5.86934E-06 |
| CYFIP2 | 1.128479 | 8.03546 | 1.18104E-09 | 2.66873E-07 |
| SLC15A3 | 1.114943 | 10.1471 | 1.27134E-07 | 8.51189E-06 |
| TNFAIP6 | 1.112458 | 9.18997 | 1.30104E-09 | 2.83851E-07 |
| SHC4 | 1.109006 | 7.93164 | 8.33022E-08 | 6.20062E-06 |
| ANGPT1 | 1.102674 | 9.31591 | 7.74866E-10 | 1.94161E-07 |
| C8ORF4 | 1.101206 | 8.51551 | 2.73076E-09 | 4.79931E-07 |
| STAT1 | 1.097489 | 12.232 | 7.3166E-08 | 5.66264E-06 |
| HS.371609 | 1.089676 | 11.0878 | 1.5348E-09 | 3.21014E-07 |
| COBLL1 | 1.085026 | 10.0957 | 2.82369E-07 | 1.58805E-05 |
| ASB5 | 1.083116 | 7.93703 | 6.33205E-08 | 5.02356E-06 |
| OKL38 | 1.080161 | 8.99374 | 1.96406E-08 | 2.08274E-06 |
| SNORD13 | 1.078067 | 9.77486 | 1.02461E-05 | 0.000249095 |
| LOC730743 | 1.077835 | 7.99452 | 3.34695E-09 | 5.64697E-07 |
| PRIC285 | 1.0762 | 9.94692 | 8.56986E-08 | 6.32321E-06 |
| CCL2 | 1.073573 | 12.926 | 5.8124E-08 | 4.68472E-06 |
| PSG3 | 1.069346 | 12.3153 | 3.17131E-08 | 3.01728E-06 |
| ADAMTS19 | 1.0688 | 7.73358 | 1.77864E-07 | 1.10872E-05 |
| PDE4B | 1.057765 | 8.3175 | 4.44722E-07 | 2.22595E-05 |
| ID2 | 1.046616 | 11.1845 | 2.92204E-09 | 5.08333E-07 |
| CYP1B1 | 1.032843 | 9.91796 | 0.001083768 | 0.008510085 |
| STAT1 | 1.032649 | 11.6745 | 9.5068E-07 | 4.02939E-05 |
| CFLAR | 1.026009 | 9.1127 | 8.59898E-07 | 3.73922E-05 |
| KIAA1644 | 1.023928 | 10.5064 | 6.17226E-06 | 0.000167965 |
| DUSP6 | 1.021102 | 8.22626 | 1.66617E-08 | 1.85759E-06 |
| LOC642333 | 1.019624 | 9.01105 | 1.50157E-06 | 5.75785E-05 |
| VGLL4 | 1.018364 | 10.144 | 8.48547E-09 | 1.13021E-06 |
| CHST7 | 1.014902 | 9.68305 | 4.75981E-09 | 7.50554E-07 |
| CXCL10 | 1.006187 | 7.93704 | 0.000476217 | 0.004520663 |
| SNCA | 1.005904 | 7.80208 | 1.09133E-06 | 4.45475E-05 |
| SEPP1 | 1.004631 | 10.192 | 6.95658E-07 | 3.1665E-05 |
| RGS11 | 1.002621 | 8.46938 | 3.70325E-08 | 3.34784E-06 |
| FGF7 | 1.001235 | 9.17908 | 1.24115E-05 | 0.000285815 |
