# Supplementary material for: Senescence‐associated tissue microenvironment promotes colon cancer formation through the secretory factor GDF15
Source: Aging Cell. 2019 Aug 6;18(6):e13013. doi: 10.1111/acel.13013 (PMC6826139; doi:10.1111/acel.13013)
Supplement: Supplementary file 3 [file ACEL-18-e13013-s003.docx]

**Supporting Methods**

**Cell culture conditions**

CCD-18Co cells and primary human fibroblast cells (s1005395, s1005379 and s1005405) were cultured in EMEM supplemented with 10% fetal bovine serum (FBS) in low (3%) O_2_. LT97 cells were cultured as previously described (Markowitz, 1994). AA/C1 and HEK293 cells were cultured in DMEM with 10% FBS. Caco-2 were cultured in [EMEM Medium](https://www.sigmaaldrich.com/catalog/product/sigma/m8403?lang=en&region=US) with 10% FBS. HT-29 were cultured in [McCoy's 5A Medium](https://www.sigmaaldrich.com/catalog/product/sigma/m8403?lang=en&region=US) with 10% FBS.

**Isolation of colon fibroblasts**

The colon tissue was cut into small (2-3mm) fragments and washed with cold DPBS three times. The tissue fragments were transferred into a 50ml conical tube and incubated with 25 ml HBSS (Ca^-^ and Mg^-^) with 5 mM EDTA at 37 ºC in shaking air bath (250 rpm) for 1.5 hours. Next the fragments were washed with cold DPBS and incubated with 20 ml HBSS (Ca^-^ and Mg^-^) with 2000 U of collagenase D and 20 U of Dispase at 37 ºC in a shaking air bath (250rpm) for 1 hour. The isolated cells were centrifuged (200x g at 4 ºC for 5min) and resuspended with EMEM medium. The resuspended cells were strained through a 70 µm strainer and incubated in TC-treated dishes at 37 ºC. After overnight incubation, the cells were washed gently with EMEM medium. The adherent cells include epithelial cells and fibroblasts at this time with only the fibroblast cells surviving the first passage. Fibroblast cells were validated as vimentin positive and EpCAM negative by immunofluorescence.

**Isolation and cell culture of human colon organoids**

The muscle and submucosa were removed when relevant and the colon tissue was cut into fragments (~2-3 mm diameter). The fragments were washed with cold DPBS three times. Next, the tissue fragments were incubated with DPBS (Ca^2+^ and Mg^2+^ free) with 8 mM EDTA and 10mM DTT with gentle shaking at 4 ºC for 1.5 hour. The fragments were transferred to a 15ml conical tube and washed with cold Advanced DMEM/F12 medium. The fragments were then vigorously shaken 10-15 times to isolate the crypts. The shaking process was repeated 4-5 times and the fractions with intact crypts were selected for organoid culture. The isolated crypts were embedded in Matrigel on ice and seeded in pre-warmed 24-well plates (add 30 μl in the center of each well). After Matrigel was polymerized (incubated at 37°C for 10 minutes), 500 μl colon organoid growth medium was added per well.

The growth medium contains Advanced DMEM/F12 medium, Wnt3a conditioned medium (Wnt 3a at 100 ng/ml), Rspondin conditioned medium (Rspondin at 1 µg/ml), Noggin conditioned medium (Noggin at 100 ng/ml), 10 mmol/L HEPES (Thermo Fisher Scientific), 1 x B27 (Thermo Fisher Scientific), 1 x N2 (Thermo Fisher Scientific), 2 mM GlutaMax (Thermo Fisher Scientific), 1 x Penicillin/Streptomycin (Thermo Fisher Scientific), 10 mM Nicotinamide (Sigma-Aldrich), 1 mM N-Acetylcysteine (Sigma-Aldrich), 50 ng/ml mouse recombinant EGF (Thermo Fisher Scientific), 10 μM SB202199 (Sigma-Aldrich), 10 nM [Leu15]-Gastrin I (Sigma-Aldrich) and 500 nM A-83-01 (Tocris).

**Cellular senescence inducement**

The protocol was modified from method described previously ({Chen, 2007 #45}). Briefly, at day 0, colon fibroblasts were seeded at a density of 5 x 10^5^ per dish in 10 cm dishes and allowed to attach for 24 hours. At day 1, the medium was removed and replaced with 400 µM H_2_O_2_ in growth medium (10 ml per dish). After 2 hours treatment, the cells were washed with DPBS 3x to remove the residual H_2_O_2_ and re-feeded with regular growth medium. The treatment was repeated at day 4 and day 7. The cell morphological changes were observed daily and cells were split in a 1:2 ratio if the confluency is > 90%. The senescence was identified by SA-β-gal staining.

**Colon epithelial cells-fibroblasts co-culture**

The colon epithelial cells-fibroblasts co-culture was performed using the Corning^®^ HTS Transwell^®^ – 96 well permeable support (Corning, cat# CL S3381). The detailed protocol is provided in Supplemental Methods. Briefly, colon epithelial cells were plated at a density of 5×10^3^ cells per well onto 96-well plates and colon fibroblast cells were plated at a density of 2×10^3^ cells per insert onto the insert for a duration of 96 hours. The Corning^®^ Transwell system-24 well permeable support (Corning, cat# CL S3396) was also used for co-culturing human colon organoids with fibroblasts. Briefly, 2 × 10^4^ fibroblast cells were plated onto 24-well plate and allowed to attach overnight. In the next day, four wells of organoids were collected (~ 50% confluency in 24-well plate) and re-suspended in 100 µl Matrigel. The Matrigel was added into 6.5 mm Transwell with 0.4 µm pore (Corining, cat# CL S3470). After Matrigel polymerized, the insert was placed into the 24-well plate with pre-seeded fibroblast cells. The fibroblast medium was aspirated and replaced with 1 ml organoids medium. 0.5 ml organoids medium was added into the insert. The organoids and fibroblast cells were co-cultured for 7 days. Medium were refreshed every 2 days. The organoids were collected for immunofluorescence for Ki67.

**Cell migration assay**

Cell motility was measured on FluoroBlok Inserts with 8 µm pores (Corning, cat# 351152). Briefly, certain number of colon epithelial cells (3 × 10^5^ cells / insert for LT-97 and AA/C1, 5 x 10^5^ cells / insert for Caco-2 and HT-29) were plated onto inserts. For co-culture experiment, 2× 10^4^ fibroblasts were plated onto 24-well plate. After 24 hours, inserts were removed and stained with DAPI (4′,6-diamidino-2-phenylindole). Membrane filters were imaged on a Nikon Elapse E300 Microscope using a 20× objective. Three representative fields were counted from each experimental group.

**Cell invasion assay**

The cell invasion assay was performed according to the protocol developed by Corning <https://www.corning.com/worldwide/en/products/life-sciences/keymatch/transwell-assay-protocol.html>. Briefly, certain number of colon epithelial cells (5 × 10^5^ cells / well for LT-97, AA/C1, Caco-2 and HT-29) were plated onto the inserts (Corning, cat# 351152) that were pre-coated with Matrigel (Corning, cat# 356231, Bedford, MA, USA) with 100µL of 300µg/mL at 4°C overnight. Uncoated inserts were used as controls for each group. For co-culture experiment, 2× 10^4^ fibroblast were plated onto 24-well plate. After 48 hours, inserts were removed and stained with DAPI. Membrane filters were imaged on a Nikon Elapse E300 Microscope using a 20× objective. Three representative fields were counted from each experimental group. The percent invasion was determined as:

$$\% invasion=\frac{mean number of cells invading through Matrigel coated insert}{mean number of cells migrating through uncoated insert}$$

**GDF15 knockdown**

The plasmids containing shGDF15 were provided by Genomics Shared Resource Core at the Fred Hutchinson Research Center (FHCRC, Seattle, WA). Lentivirus were produced using HEK293 cells transfected with shGDF15 individual constructs and packaging vectors (pMD2.G and psPAZ2) (addgene, Cambridge, MA, USA). The pGIPZ vector with scrambled shRNA was used as a negative control plasmid. Lentiviral particles were collected at 24 and 48 hours after transfection. CCD-18Co cells or primary fibroblast cells were transduced with lentiviral particles. Seven days after transduction, the cells were selected by incubation with 0.8 µg/ml puromycin (Thermo Fisher Scientific).

**Immunofluorescence studies of human colon specimens**

## Immunofluorescent staining was performed using 5 µm sections of formalin fixed, paraffin embedded specimens. Following paraffin removal and rehydration of tissues, sections were steam treated for Dako Target Retrieval Solution, Citrate pH 6.0. Samples were blocked in TCT buffer (50 mM Tris pH 7.6, 150 mM NaCl, 0.25% Casein, 0.1% Tween20) with 10% Human serum. Specimens were then incubated with primary antibodies diluted in TBS with 1% BSA for 1 hour. Anti-γH2A.X (Millipore, cat# 05-636) was used at 1:50 and anti-Ki67 (Novacastra, cat# NCL-Ki67p) was used at 1:50. Then the sections were incubated with secondary antibodies and tertiary antibodies and counterstained with DAPI for 5 minutes. The secondary antibodies include goat anti-mouse secondary antibody at 1:100 and biotin-goat anti-rabbit antibody at 1:200 in TBS with 1% BSA for 1 hour. Tertiary antibodies include SA-Alexa568 and SA-Alexa647 in TBS with 1% BSA at 1:500. Fluorescent images were acquired using TissueFAX (Tissuegnostics Vienna, Austria) automated fluorescent microscope at 10X magnification. Confocal fluorescent images were acquired using Zeiss LSM (laser scanning microscope) 780 confocal microscope equipped with a 20x and 63× oil immersion lens.

TissueQuest (Tissuegnostics Vienna, Austria) image analysis software was used for the quantification of senescent cells. Detection thresholds were set within the TissueQuest software for each fluorescent channel to account for background levels of fluorescence on a batch by batch basis. Non-specific staining was corrected for using a DAPI positive nuclear mask. Regions of interest were drawn on the acquired images to restrict analysis to stromal tissue only. Based on pre-set master channel parameters, DAPI was used as a master marker (nucleus) to identify single cells. The intensity of staining of γH2A.X within nucleus mask was quantified. The mean intensity of γH2A.X was plotted against mean DAPI intensity. γH2A.X positive cells were identified based on the threshold values that were set by engaging negative isotype controls. Ki67 positive cells were identified using the same strategy. The percentage of senescent cells was quantified as:

$$\% senescent cells=\frac{number of \gamma H2A.X\left( + \right) cells-number of both Ki67\left( + \right) and \gamma H2A.X\left( + \right) cells}{total cells}$$

**Immunofluorescence studies of human colon organoids**

The culture medium was removed and the organoids were collected by gently pipetting Matrigel in 0.5 ml ice-cold DPBS with a cut tip and transferred to a 1.5 ml Eppendorf tube. The organoids were centrifuged at 150x g at 4 ºC for 5 minutes. After aspirating the supernatant, organoids were fixed at room temperature for 1 hour in 4% paraformaldehyde (Sigma, Saint Louis, MO, USA). The organoids pellets were re-suspended in 200 µl pre-melted HistoGel (Fisher Scientific, Runcorn, Cheshire, UK) by gently pipetting up and down using a cut tip. The HistoGel was added onto a Parafilm as a droplet and allowed to cool for 30 minutes. The solidified organoids embedded HistoGel was transferred to 70% ethanol and processed for paraffin embedding. The immunofluorescent staining was performed using 5 µm sections of FFPE organoids samples following routine protocols. Following paraffin removal and rehydration of tissues, sections were steam treated for antigen retrieval using Dako Target Retrieval Solution, Citrate pH 6.0. Samples were blocked in TCT buffer (50 mM Tris pH 7.6, 150 mM NaCl, 0.25% Casein, 0.1% Tween20) with 10% Human serum. Specimens were then incubated with anti-Ki67 (Novacastra, cat# NCL-Ki67p) antibody diluted in TBS + 1% BSA at 1:50 for 1 hour. Then the sections were incubated with goat anti-mouse secondary antibody and tertiary antibodies and counterstained with DAPI. Fluorescent images were acquired using Nikon Eclipse E800 fluorescent microscope at 20X magnification. Image analysis methods were performed using TissueQuest^TM^ software. Based on pre-set master channel parameters, DAPI was used as a master marker (nucleus) to identify single cells. The intensity of staining of Ki67 within nucleus mask was quantified. The mean intensity of staining was plotted against mean DAPI intensity. Positive cells were identified based on the threshold values that were set by engaging negative isotype controls. The percentage of Ki67 positive cells in each organoid was quantified.

**Immunofluorescence studies of cell lines and primary cells**

Cells were allowed to grow on coverslip overnight and fixed with 4% paraformaldehyde for 10 min. Fixed cells were permeabilized with 0.1% Triton-X 100 and blocked in 5% Goat Serum (Invitrogen). Cells were stained for anti-vimentin (Cell Signaling Technologies, cat# 9855) antibody at 1:50 and anti-EpCAM (Cell Signaling Technologies, cat# 3199) antibody at 1:500 in 1% BSA at 4 ºC overnight and counterstained with DAPI. Fluorescent images were acquired using Nikon Eclipse E800 fluorescent microscope at 20X magnification.

**Western blotting**

Treated cells were washed with ice cold PBS and lysed with RIPA buffer (ThermoFisher Scientific, cat# 89900) supplemented with cOmplete^TM^, Mini Protease Inhibitor Cocktail (Sigma, cat# 11836153001), phosphatase inhibitor cocktail 2 (Sigma, cat#P5726) and phosphatase inhibitor cocktail 3 (Sigma, cat# P0044). Cell lysates were processed for SDS-PAGE, transferred to polyvinylidene fluoride membranes and individual proteins were detected with specific antibodies. The following commercial antibodies were used: anti-GDF15 (abcam, cat#39999), anti-phospho Akt (Ser473) (Cell Signaling Technology, cat# 9271), anti-Akt (Cell Signaling Technology, cat# 4691), anti-phospho p38 MAPK (Thr180/Tyr182) (Cell Signaling Technology, cat# 4511), anti- p38 MAPK (Cell Signaling Technology, cat# 8690), anti-phospho Erk(1/2) (Thr202/Tyr204) (Cell Signaling Technology, cat# 4370), anti- Erk(1/2) (Cell Signaling Technology, cat# 4695), anti-phospho Smad2 (Ser465/467) (Cell Signaling Technology, cat# 3108) and anti-GAPDH (Cell Signaling Technology, cat# 3683). The positive and negative control lysates were used as below, p39 MAP Kinase Control (Cell Signaling Technology, cat#9213), Akt Control (Cell Signaling Technology, cat#9273), and p44/42 MAPK (erk1/2) Control (Cell Signaling Technology, cat#9194). EGF positive and negative cell lysates generated following methods has been previously described(Yu et al., 2018).

**RNA extraction from cell lines and primary tissues**

For cell lines and primary cells, total mRNA was extracted from cells using TRIzol Reagent (Thermo Fisher Scientific) according to the manufacturer’s protocols. For primary tissue samples, total RNA was extracted using the RNeasy Mini Kit (Qiagen, Hilden, Germany) following manufacture’s instructions. RNA integrity was assessed using the Agilent 2200 TapeStation System (Agilent Technologies, Santa Clara, CA, USA).

**Quantitative real-time PCR**

RNA concentrations were measured at 260 nm using NanoDrop (Thermo Fisher Scientific). The cDNA was synthesized using iScript^TM^ cDNA Synthesis Kit (Bio-rad, Hercules, CA, USA) according to manufacturer’s instructions. Quantitative real-time quantitative PCR was performed using SYBR Green Master Mix (Bio-Rad, Hercules, CA, USA). Primers sequences for GDF15 are forward 5′-GGCCAACCAGAGCTGGGAAG-3′; reverse 5′-GCCCGAGAGATAC-GCAGGTG-3′. Primers for GAPDH are forward 5’ -TGCACCACCAACTGCTTAG -3’, reverse 5’-GATGCAGGGATGATGTTC-3’. GAPDH expression values were used for normalization of cDNA loading between samples. All quantitative real-time PCR were performed via a CFX96 Touch Real-Time PCR Detection System (BioRad) and results were analyzed using CFX Manager software, version 3.1 (BioRad).

**Spheroid generation**

Spheroid cell culture was modified from methods previously described in (Poudel et al., 2018). In short, HT 29 cells were trypsinzed, counted, and resuspended in 1x10^3^ cells per 12uL McCoy 5a media (+10% FBS and +1% penicillin). Pipette 12uL drops of cell suspension onto a lid of a 10cm petri dish. Hang droplets upside down in the 10cm petri dish humidified by soaking kimwipes in PBS and incubate at 37°C for 72 hours. After 72 hours, precoat a 48 well plate with 120uL of Matrigel (Corning, cat# 356231, Bedford, MA, USA), incubate at 37°C for 30 minutes. Gently pipette the spheroids into 100uL of a 50% (vol/vol) Matrigel to Collagen I, Rat Tail (Corning, cat #354236, Bedford, MA, USA) solution and layer on top of hardened Matrigel. Incubate at 37°C for 10 minutes and add 200uL McCoy 5a media to prevent dry out.

**Spheroid Invasion Assay**

Spheroids were treated with 50% (vol/vol) of McCoy 5a and senescent CCD18 conditioned media or a 50% (vol/vol) of McCoy 5a and EMEM, as a control group. Spheroids were monitored over a one-week period and spheroid images were imaged on a Nikon Elapse E300 Microscope using the 10x objective. Measurements of spheroid invasion was preformed using Image J.

**Correlation of GDF15 expression with patient age in the TCGA-COAD data.**

The correlation analysis was performed using RNA-seq datasets from 40 normal and 271 primary colon cancer tissue (COAD) samples obtained from the TCGA (<https://tcga-data.nci.nih.gov/tcga>). The detailed analysis is provided in Supplemental Methods. Original RNA expression values (normalized read counts) were used for the downstream analyses. The normalized RNA-Seq (By Illumina HiSeq platform) counts of all genes from the legacy database were aligned against the hg19 reference genome and clinical information of samples using the *TCGAbiolinks* R package. After data cleaning, 40 normal and 271 primary colon cancer tissue (COAD) samples with their tumor stage and age annotations remained and were used for the relevant studies.

**References:**

Markowitz, S. D., Myeroff, L., Cooper, M. J., Traicoff, J., Kochera, M., Lutterbaugh, J., . . . Willson, J. K. (1994). A benign cultured colon adenoma bears three genetically altered colon cancer oncogenes, but progresses to tumorigenicity and transforming growth factor-beta independence without inactivating the p53 tumor suppressor gene. *J Clin Invest, 93*(3), 1005-1013. doi:10.1172/JCI117048

Poudel, K. R., Roh-Johnson, M., Su, A., Ho, T., Mathsyaraja, H., Anderson, S., . . . Bai, J. (2018). Competition between TIAM1 and Membranes Balances Endophilin A3 Activity in Cancer Metastasis. *Dev Cell, 45*(6), 738-752 e736. doi:10.1016/j.devcel.2018.05.021

Yu, M., Maden, S. K., Stachler, M., Kaz, A. M., Ayers, J., Guo, Y., . . . Grady, W. M. (2018). Subtypes of Barrett's oesophagus and oesophageal adenocarcinoma based on genome-wide methylation analysis. *Gut*. doi:10.1136/gutjnl-2017-314544
